# Supplementary material for: Soma-localized Rab39 inhibits synaptic autophagy by controlling trafficking of Atg9 vesicles
Source: EMBO J. 2025 Aug 21;44(20):5662–93. doi: 10.1038/s44318-025-00536-8 (PMC12528412; doi:10.1038/s44318-025-00536-8)
Supplement: Supplementary file 1 — Appendix [file 44318_2025_536_MOESM1_ESM.pdf]

Appendix for:

Soma-localized Rab39 inhibits synaptic autophagy by controlling trafficking of Atg9 vesicles

Ayse Kilic[1,2], Gokhan Ozturan[1,2], Dirk Vandekerkhove[1,2], Sabine Kuenen[1,2], Jef Swerts[1,2], Esther Muñoz Pedrazo[1,2], Carles Calatayud Aristoy[1,2], Abril Escamilla Ayala[3], Nikky Corthout[3], Pablo Hernández Varas[3], Stephane Plaisance[4], Valerie Uytterhoeven[1,2], Eliana Nachman[1,2*], Patrik Verstreken[1,2,*]*

[1] VIB-KU Leuven Center for Brain & Disease Research, 3000 Leuven, Belgium.

[2] KU Leuven, Department of Neurosciences, Leuven Brain Institute, 3000 Leuven, Belgium.

[3] VIB BioImaging Core, VIB-Center for Brain and Disease Research, 3000 Leuven, Belgium.

[4] VIB Nucleomics Core, 3000 Leuven, Belgium

[*]Co-corresponding authors: valerie.uytterhoeven@kuleuven.be, eliana.nachman@kuleuven.be, patrik.verstreken@kuleuven.be

Table of contents:

Appendix Figure S1: Autophagy is not affected in the neuronal somata of the ventral nerve cord of *rab39*^{KO} mutants p2

Appendix Figure S2: The levels of several synaptic proteins are unaltered in *rab39*^{KO} animals p3

Appendix Table S1: Vesicle and mitochondria directionality counts p4

Appendix Table S2: *Drosophila* stocks used in this study p5

Appendix Table S3: Antibodies used in this study p6

Appendix figure S1. Autophagy is not affected in the neuronal somata of the ventral nerve cord of *rab39*^{KO} mutants

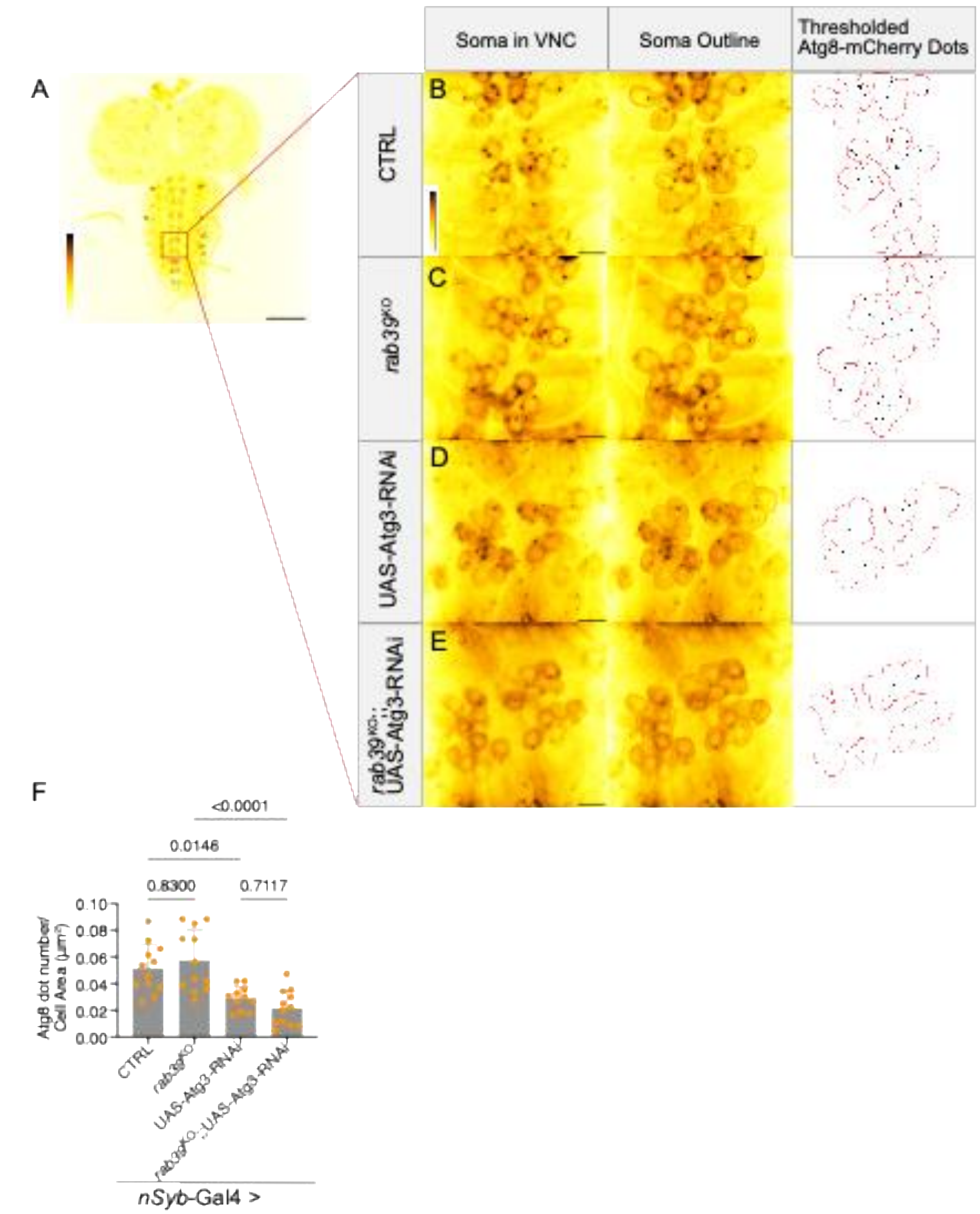

Appendix Figure S1. Autophagy is not affected in neuronal somata of the ventral nerve cord in *rab39*^{KO} mutants

(A) Representative confocal image of the larval ventral nerve cord expressing Atg8-mCherry. Fluorescence intensities are shown using the gray value range 22–2162. Scale bar: 100 µm. (B–E) Zoomed-in views of neuronal cell bodies from control ($w^{1118}w^{+}$, CTRL) (B), $rab39^{KO}$ (C), UAS-*atg3-RNAi* (D), and $rab39^{KO}$*;;UAS-atg3-RNAi* (E) animals, with pan-neuronal expression driven by *nSyb*-Gal4. Fluorescence intensities are shown using the gray value range 89–609. Neuronal cell bodies were thresholded and outlined in red; Atg8-mCherry puncta were marked for particle counting. Scale bar: 10 µm. (F) Quantification of Atg8-mCherry puncta per cell area from the experiment shown in (B–E) $rab39^{KO}$ - $rab39^{KO}$*;;UAS-atg3-RNAi exact* $p = 3.80837329 \times 10^{-5}$. Statistical test: one-way ANOVA with Tukey's multiple comparison test; $n = 12$; error bars: mean ± SD.

Appendix figure S2. The levels of several synaptic proteins are unaltered in *rab39*^{KO} animals

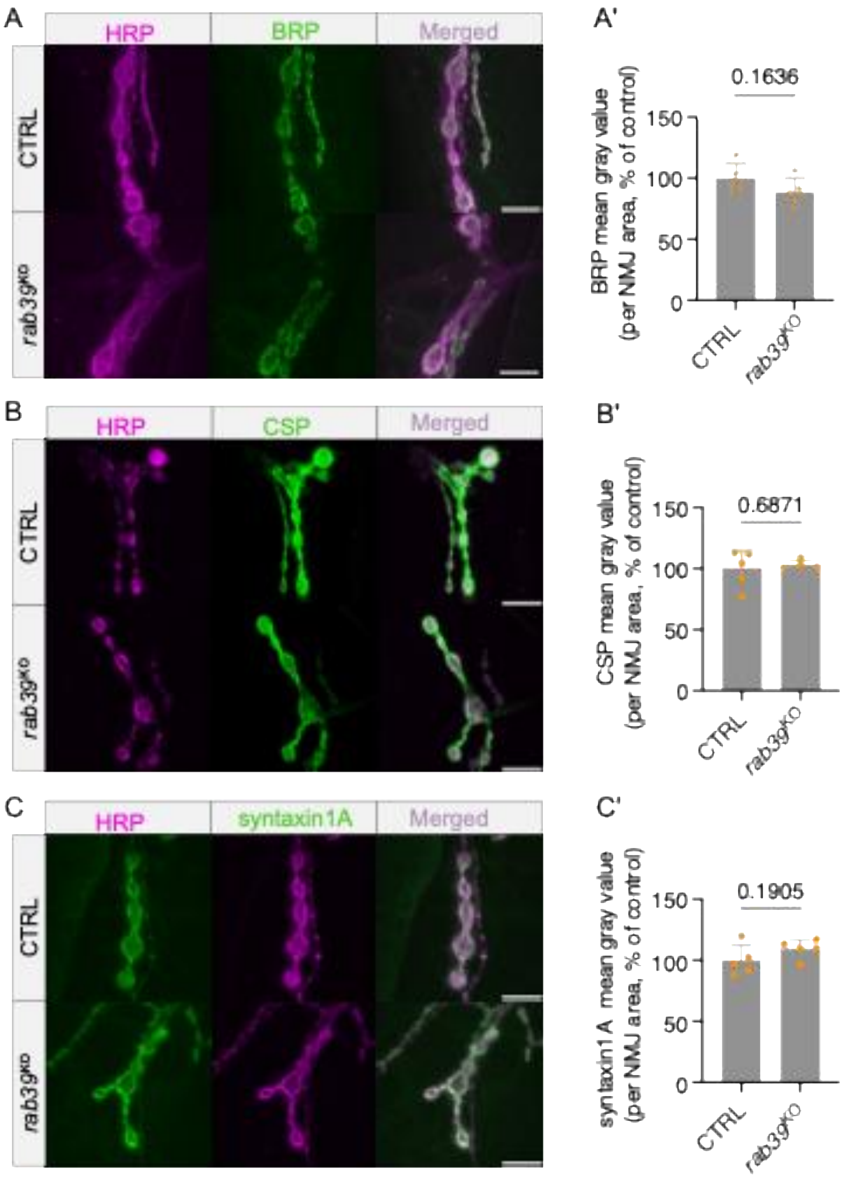

Appendix Figure S2. The levels of several synaptic proteins are unaltered in *rab39*^{KO} animals
(A–C) Representative maximum intensity projection confocal images of NMJ boutons from control ($w^{1118}w^{+}$, CTRL) and *rab39*^{KO} animals. Tissues were co-stained with anti-HRP (magenta) to outline neuronal membranes and with: (A) anti-BRP (green) to label active zones, (B) anti-CSP (green) to label synaptic vesicle-associated protein, and (C) anti-Syntaxin-1A (green) to label membrane-associated protein at presynaptic sites. Scale bar: 10 µm. (A′–C′) Quantification of average pixel intensity per NMJ area (HRP-positive region) for BRP (A′), CSP (B′), and Syntaxin-1A (C′). Statistical test: unpaired Student's t-test; *n* = 5; error bars: mean ± SD.

Appendix Table S1: Vesicle and mitochondria directionality counts

Figure	Vesicle/Organelle	Genotype	Direction	Count
3J	Atg8-mcherry	CTRL	Retrograde	3
			Stationary	12
		rab39^{KO}	Retrograde	10
			Stationary	13

Figure	Vesicle/Organelle	Genotype	Direction	Count
7N	Atg9-mcherry	CTRL	Anterograde	22
			Retrograde	4
			Stationary	2
		rab39^{KO}	Anterograde	42
			Retrograde	6
			Stationary	7

Figure	Vesicle/Organelle	Genotype	Direction	Count
EV4E	mitoGFP	CTRL	Anterograde	115
			Retrograde	81
		rab39^{KO}	Anterograde	114
			Retrograde	81

Figure	Vesicle/Organelle	Genotype	Direction	Count
EV4I	syt-eGFP	CTRL	Anterograde	131
			Retrograde	204
		rab39^{KO}	Anterograde	123
			Retrograde	163

Appendix Table S2: *Drosophila* stocks used in this study

Line name	Genotype	Stock number (if relevant)	Reference (if relevant)
w^{1118}	Canton-S-iso-w1118		Kaempf et al.,2024
w^{1118}*w*$^{+}$	Canton-S-iso-M{w+} w1118		Kaempf et al.,2024
rab39^{KO}	w[1118] TI{w[+]=white-STAR}Rab39[KOWS]/FM7a		Kaempf et al.,2024
synj^{KO}	w[1118]; TI{w[+]=white-STAR}Synj[KOWS]/CyO Tb[1]		Kaempf et al.,2024
lrrk^{KO}	w[1118]; TI{w[+]=white-STAR}Lrrk[KOWS]/TM6C Tb[1] Sb[1]		Kaempf et al.,2024
hRab39B^{WT}	w1118[cs], hRab39B[WT_KI]		Pech et al.,2024
hRab39B^{T168K}	w1118[cs], hRab39B[T168K_KI]		Pech et al.,2024
endoA^{S75D}	w; endo[S75D]		Matta et al., 2012
*endoA*D4	w;;endoAD4/Tm6c		Verstreken et al., 2002
*endoA*26	w;;endoA26/Tm6b		Guichet et al.,2002
UAS-Atg8-GFP-mcherry	w;;UASmCherry-GFP::Atg8		Vanhauwaert et al.,2017
FT::nsyb	w;;UAS-FluorescentTimer-nSyb		Fernandes et al.,2014
UAS-EndoA^{DA}	w*;UAS EndoA D265A		Bademosi et al.,2023
D42-Gal4,UAS-mitoGFP	w; ; D42GAL4, UASmitoGFP / TM6b		Piling et al., 2006
D42-Gal4, UAS-GFP-Atg9	w;;D42-Gal4, UAS-GFP-Atg9		Soukup et al., 2016
shot^{3}	w[*]; P{w[+mW.hs]=FRT(w[hs])}G13 shot[3]/CyO, P{w[+mW.hs]=ase-lacZF:2.0}PK2	BDCS #5141; FBal0051150	
UAS-Atg3-Rnai	y[1] sc[*] v[1] sev[21]; P{y[+t7.7] v[+t1.8]=TRiP.HMS01348}attP2	BDSC #34359; FBal0257941	

Appendix Table S3: Antibodies used in this study

Antibody	Concentration	Supplier	Catalog number
Rabbit anti-HRP	1:2000	Jackson ImmunoResearch	323-005-021
Alexa Fluor 647-conjugated goat anti-HRP	1:100	Jackson ImmunoResearch	123-605-021
Mouse anti-DLG	1:50 or 1:100	DSHB	4F3
Mouse anti-Brp (nc82)	1:250	DSHB	NC82
Mouse anti-CSP (AB49)	1:500	DSHB	AB49
Mouse anti-Syntaxin 1A (8C3)	1:20	DSHB	8C3
Mouse anti-Rab7	1:50	DSHB	Rab7
Rat anti-Elav	1:500	DSHB	7E8A10
Rabbit anti-Lamp1	1:500	Abcam	ab24170
Rabbit anti-GM130	1:500	Abcam	ab30637
Rabbit anti-syntaxin16	1:500	Abcam	ab32340
Chicken anti-GFP	1:500	Invitrogen	A10262
Rabbit anti-mCherry	1:500	R&D (NOVUS)	NBP2-25157
Rabbit anti-TH	1:500	Sigma-Aldrich	AB152
Alexa Fluor 488 goat anti-rabbit	1:500 or 1:250	Invitrogen	A11034
Alexa Fluor 488 goat anti-chicken	1:500 or 1:250	Invitrogen	A21449
Alexa Fluor 488 goat anti-mouse IgG1	1:500 or 1:250	Invitrogen	A21121
Alexa Fluor 555 goat anti-rabbit	1:500 or 1:250	Invitrogen	A27039
Alexa Fluor 555 goat anti-mouse IgG2a	1:500 or 1:250	Invitrogen	A21137
Alexa Fluor 555 goat anti-rat	1:500 or 1:250	Invitrogen	A21434
Alexa Fluor 647 goat anti-mouse	1:500 or 1:250	Invitrogen	A21236
Alexa Fluor 647 goat anti-rat	1:500 or 1:250	Invitrogen	A21247
