# Supplementary material for: Soma-localized Rab39 inhibits synaptic autophagy by controlling trafficking of Atg9 vesicles
Source: EMBO J. 2025 Aug 21;44(20):5662–93. doi: 10.1038/s44318-025-00536-8 (PMC12528412; doi:10.1038/s44318-025-00536-8)
Supplement: Supplementary file 4 — Movie EV1 [file 44318_2025_536_MOESM4_ESM.zip › EMBOJ-2024-119885R1_Movie EV1/Legend Movie EV1.docx]

**Movie EV1. Atg9-mCherry vesicle dynamics in axons of Drosophila 3rd instar larvae (*rab39*^KO^).**
Time-lapse movie showing movement of Atg9-mCherry–positive vesicles in segmental axons of a rab39^KO^ Drosophila 3rd instar larva. Images were acquired at 6.52-second intervals for a total of 30 frames (~195.6 seconds total duration). The movie is played back at 10 frames per second, compressing the time course to ~3 seconds.
