# Supplementary material for: Soma-localized Rab39 inhibits synaptic autophagy by controlling trafficking of Atg9 vesicles
Source: EMBO J. 2025 Aug 21;44(20):5662–93. doi: 10.1038/s44318-025-00536-8 (PMC12528412; doi:10.1038/s44318-025-00536-8)
Supplement: Supplementary file 5 — Movie EV2 [file 44318_2025_536_MOESM5_ESM.zip › EMBOJ-2024-119885R1_Movie EV2/Legend Movie EV2.docx]

**Movie EV2. Atg9-mCherry vesicle dynamics in axons of Drosophila 3rd instar larvae (w^1118^w^+^).**
Same imaging and playback settings as in Movie 1, showing vesicle dynamics in a *w*^1118^ *w*^+^control larva.
