# Supplementary figures and images for: Soma-localized Rab39 inhibits synaptic autophagy by controlling trafficking of Atg9 vesicles
Source: EMBO J. 2025 Aug 21;44(20):5662–93. doi: 10.1038/s44318-025-00536-8 (PMC12528412; doi:10.1038/s44318-025-00536-8)

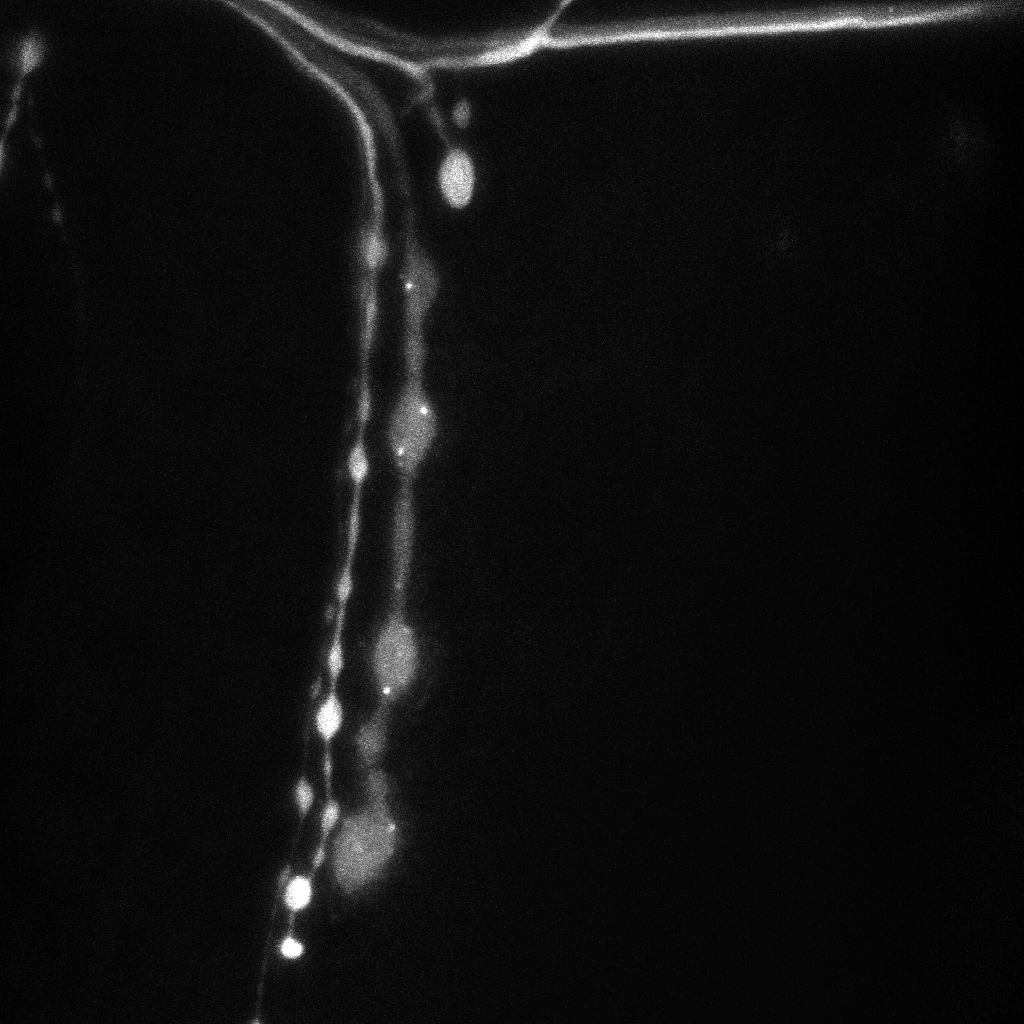

Supplement: Supplementary file 8 — Source data Fig. 3 [file 44318_2025_536_MOESM8_ESM.zip › Figure 3/Figure 3L/w1118_UASGFPmchrAtg8.tif]

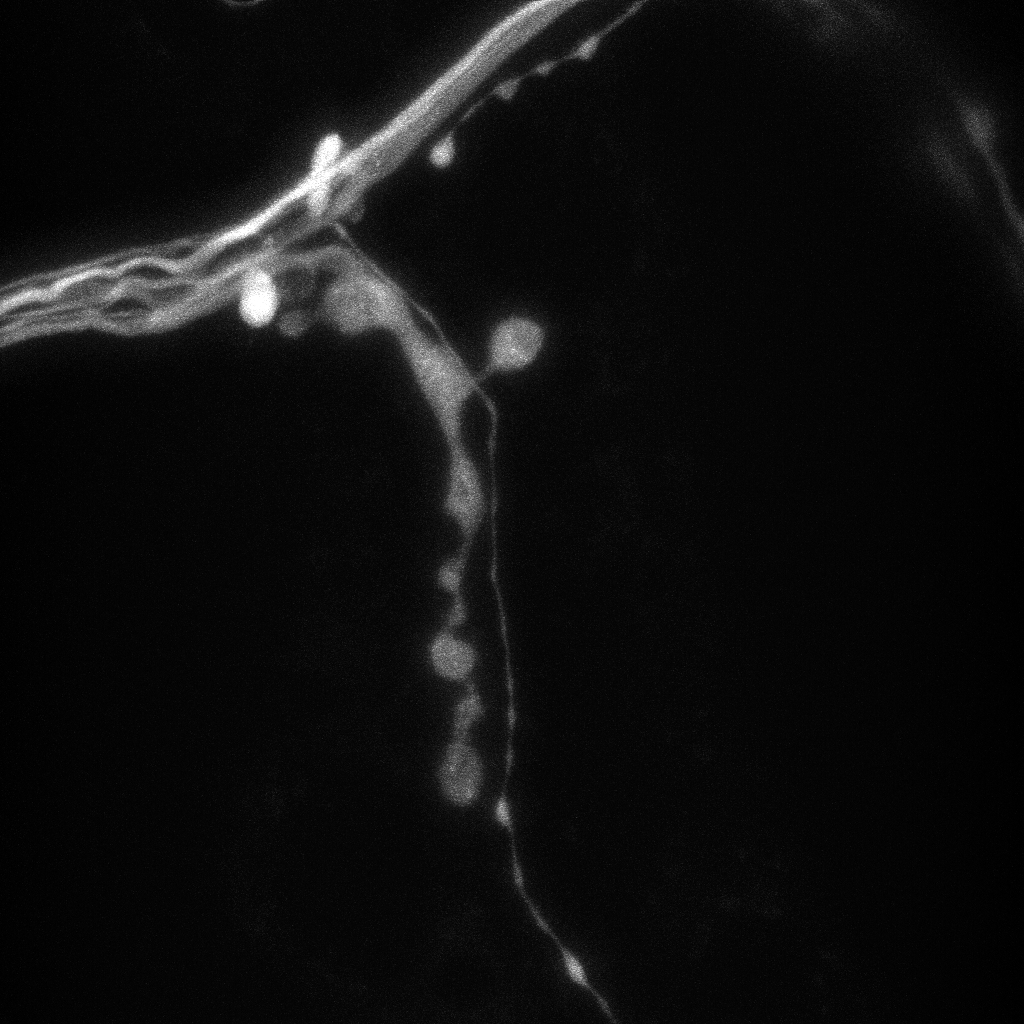

Supplement: Supplementary file 8 — Source data Fig. 3 [file 44318_2025_536_MOESM8_ESM.zip › Figure 3/Figure 3L/rab39KO_57C10_UASGFPmchrAtg8.tif]

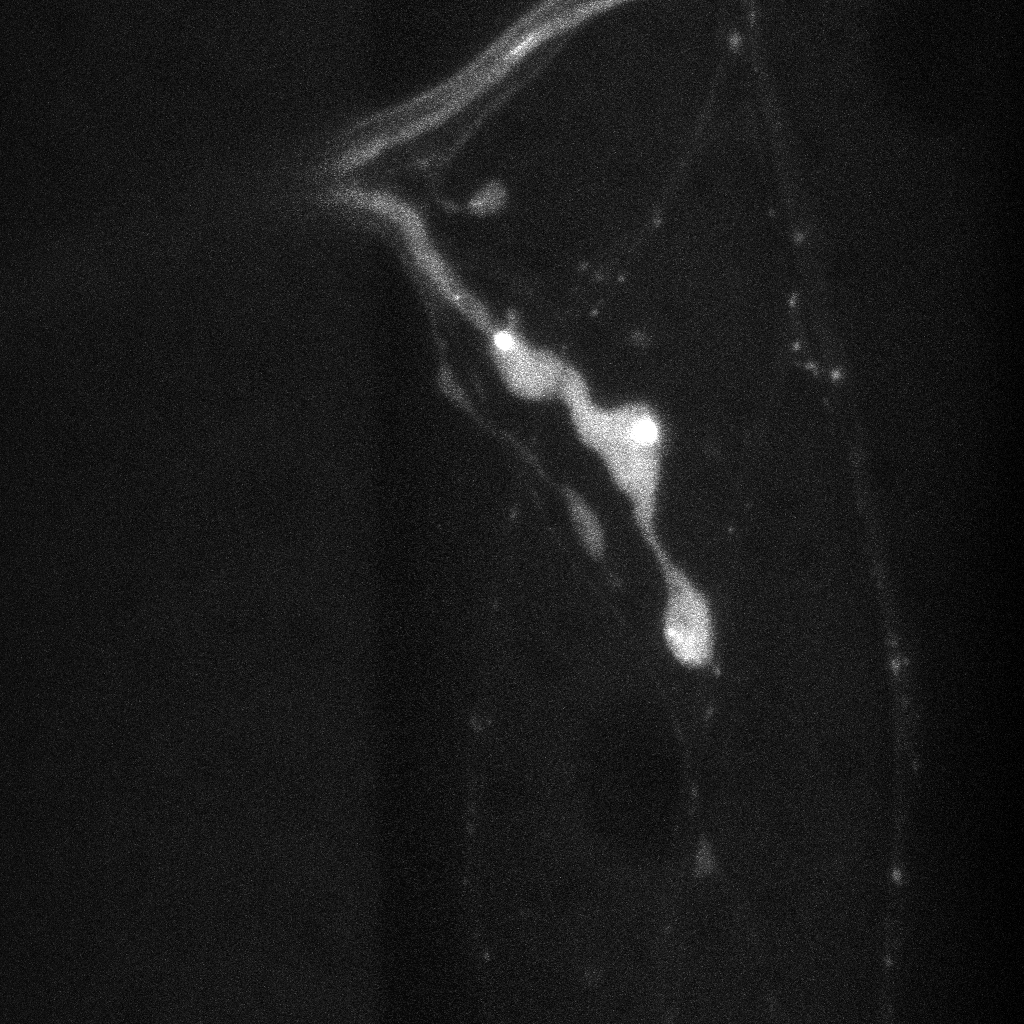

Supplement: Supplementary file 8 — Source data Fig. 3 [file 44318_2025_536_MOESM8_ESM.zip › Figure 3/Figure 3B/rab39KO.tif]

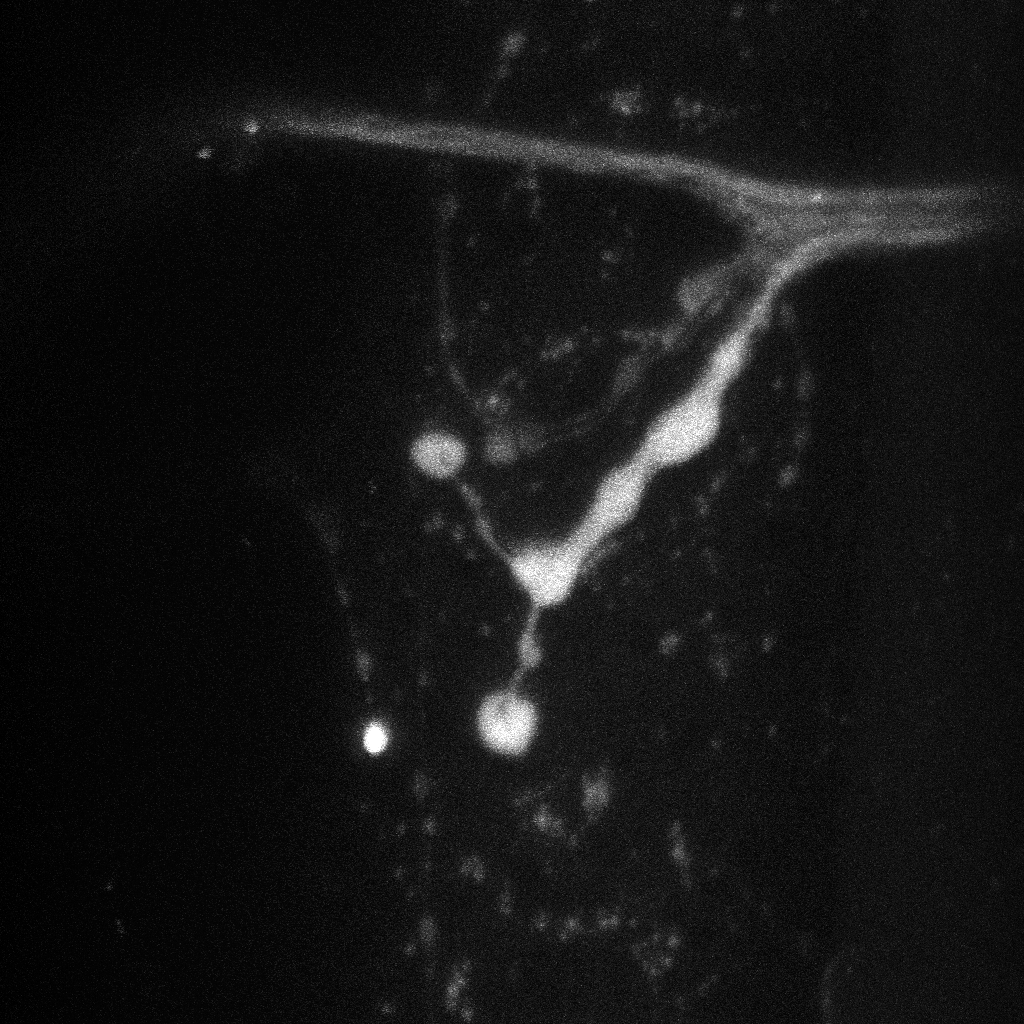

Supplement: Supplementary file 8 — Source data Fig. 3 [file 44318_2025_536_MOESM8_ESM.zip › Figure 3/Figure 3B/w1118.tif]

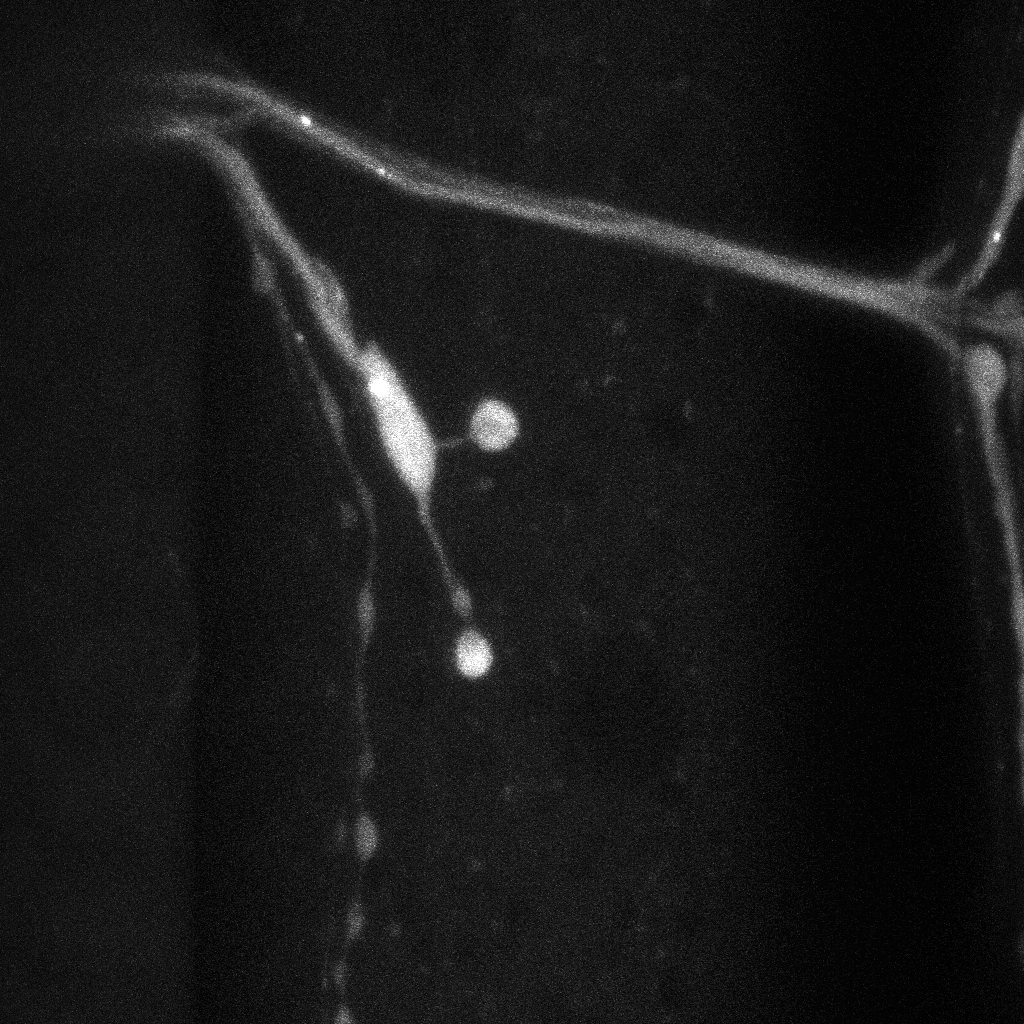

Supplement: Supplementary file 8 — Source data Fig. 3 [file 44318_2025_536_MOESM8_ESM.zip › Figure 3/Figure 3P/rab39KO.tif]

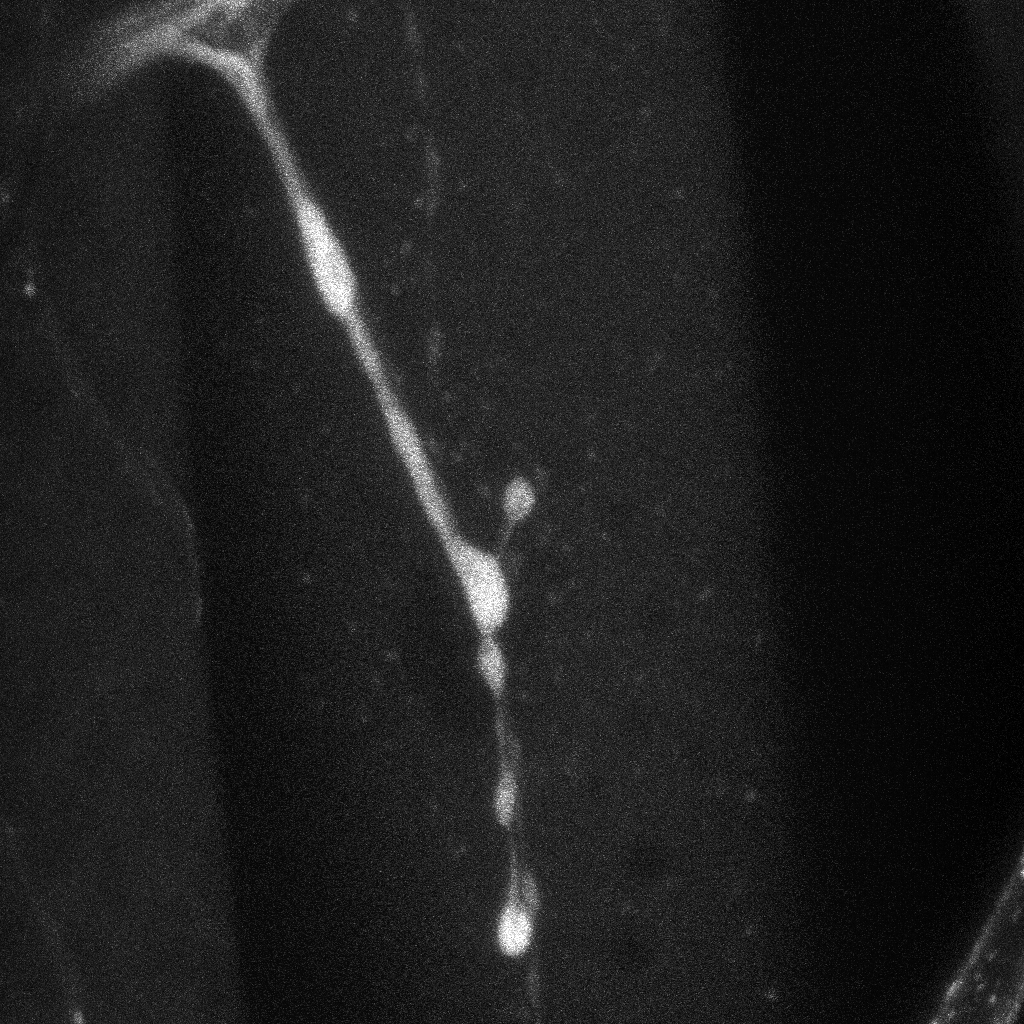

Supplement: Supplementary file 8 — Source data Fig. 3 [file 44318_2025_536_MOESM8_ESM.zip › Figure 3/Figure 3P/w1118.tif]

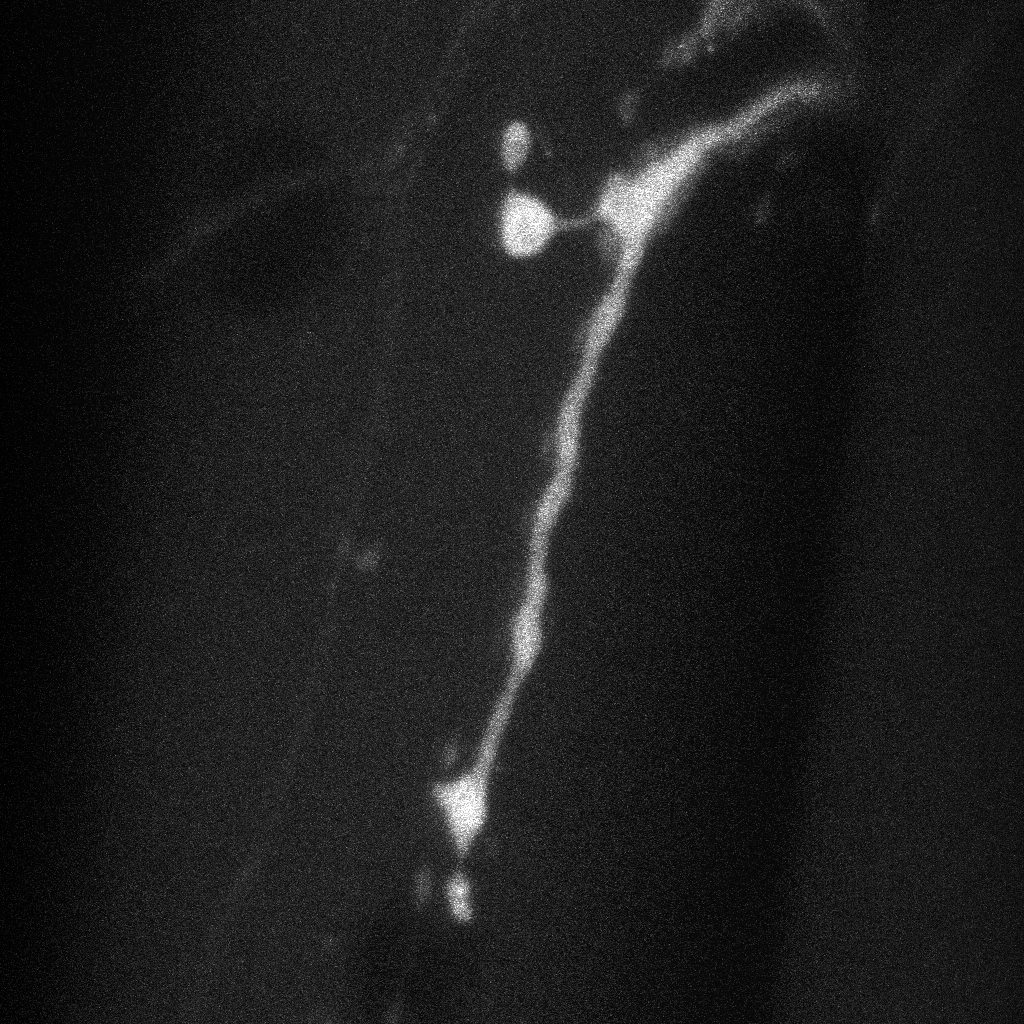

Supplement: Supplementary file 8 — Source data Fig. 3 [file 44318_2025_536_MOESM8_ESM.zip › Figure 3/Figure 3C/rab39KO_UASRab39WT.tif]

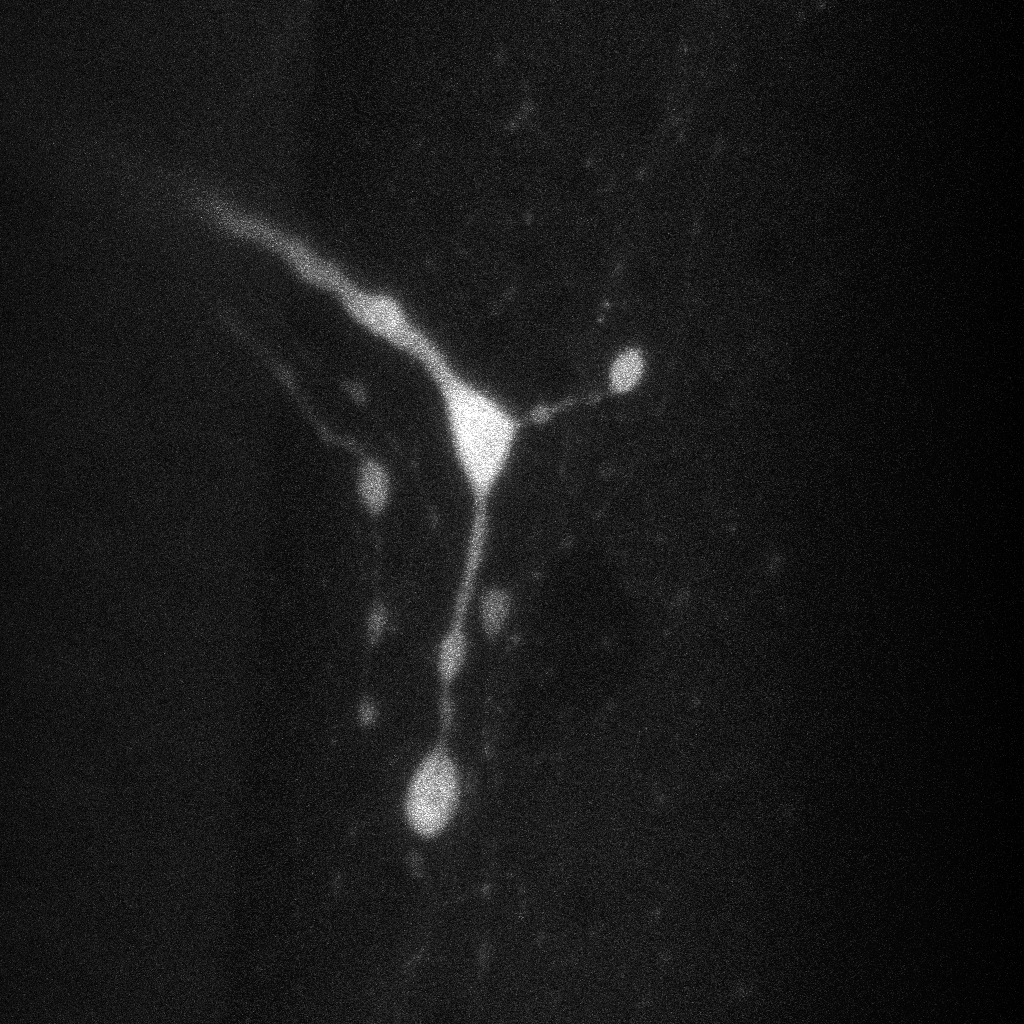

Supplement: Supplementary file 8 — Source data Fig. 3 [file 44318_2025_536_MOESM8_ESM.zip › Figure 3/Figure 3C/UASRab39WT.tif]

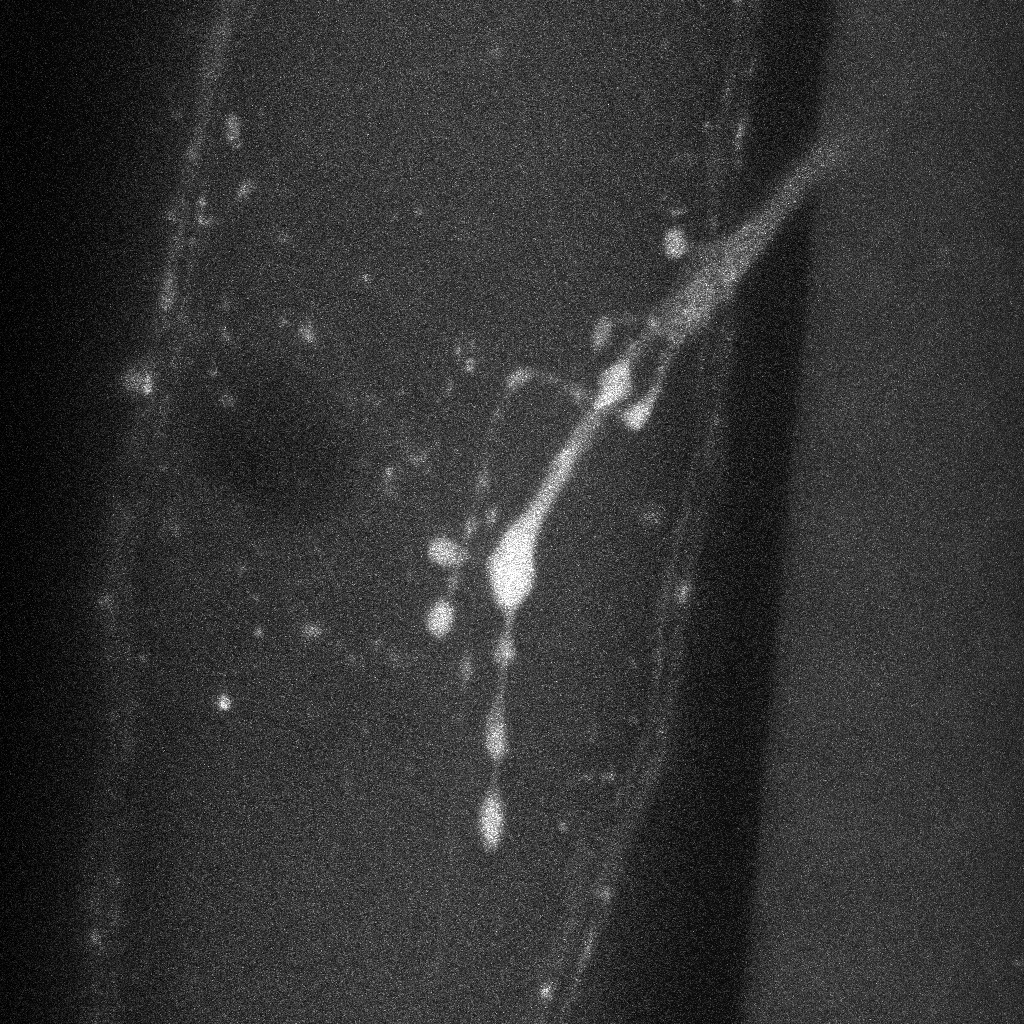

Supplement: Supplementary file 8 — Source data Fig. 3 [file 44318_2025_536_MOESM8_ESM.zip › Figure 3/Figure 3D/rab39KO_UASAtg3RNAi.tif]

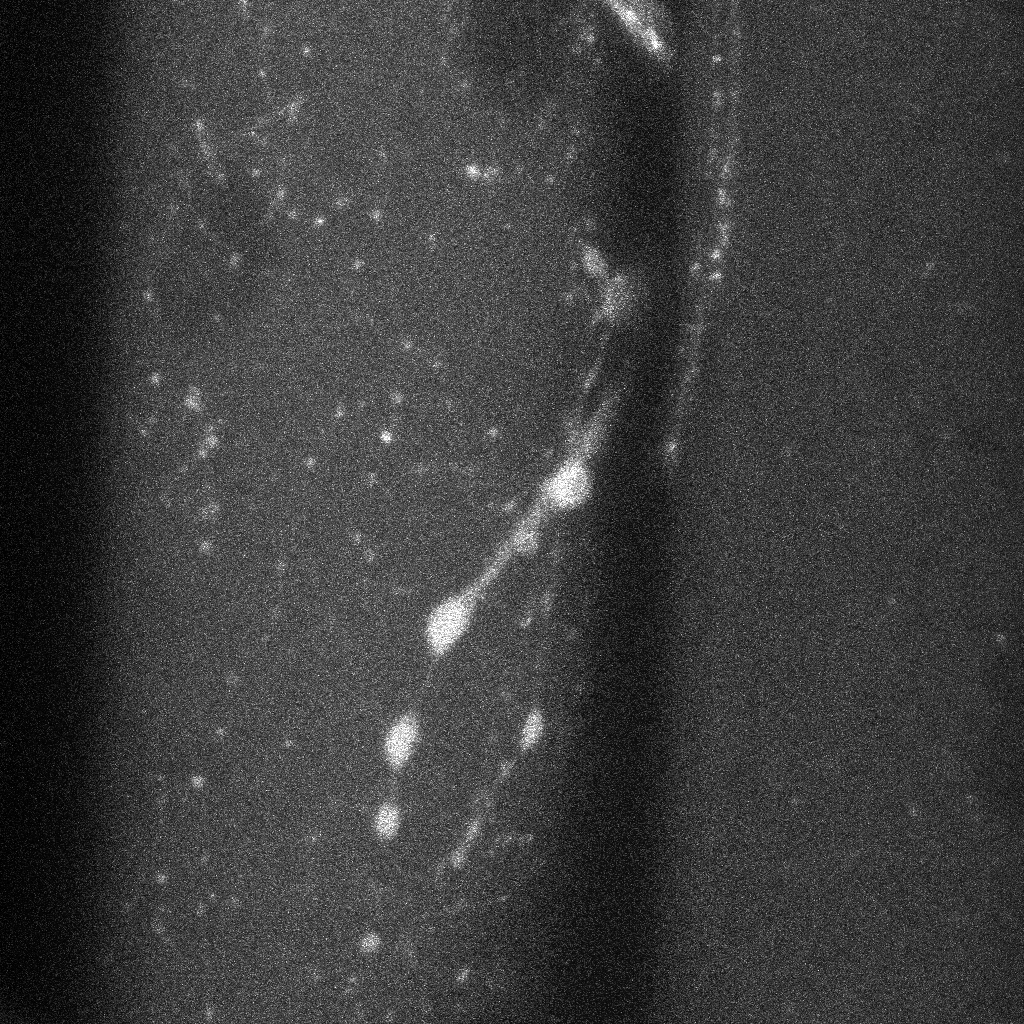

Supplement: Supplementary file 8 — Source data Fig. 3 [file 44318_2025_536_MOESM8_ESM.zip › Figure 3/Figure 3D/w1118_UASAtg3RNAi.tif]

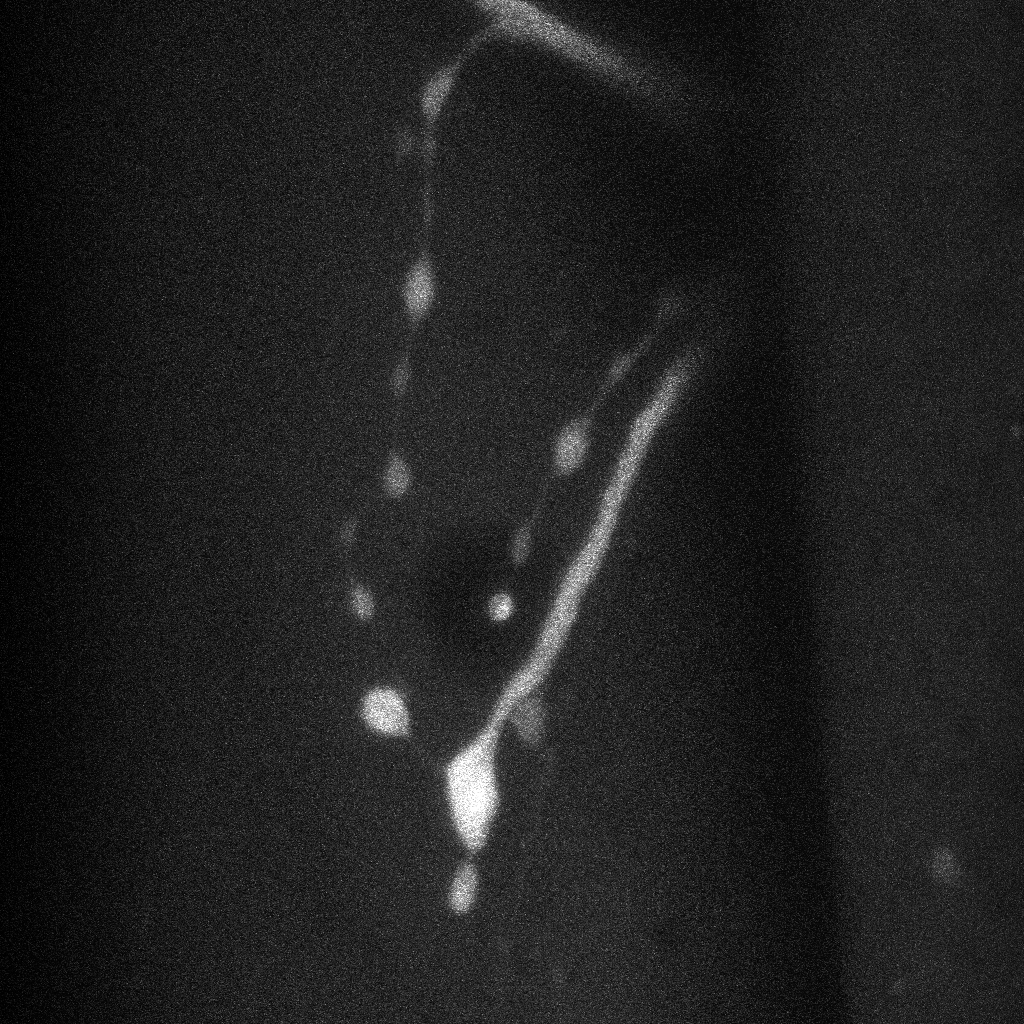

Supplement: Supplementary file 8 — Source data Fig. 3 [file 44318_2025_536_MOESM8_ESM.zip › Figure 3/Figure 3Q/rab39KO_EndoADA.tif]

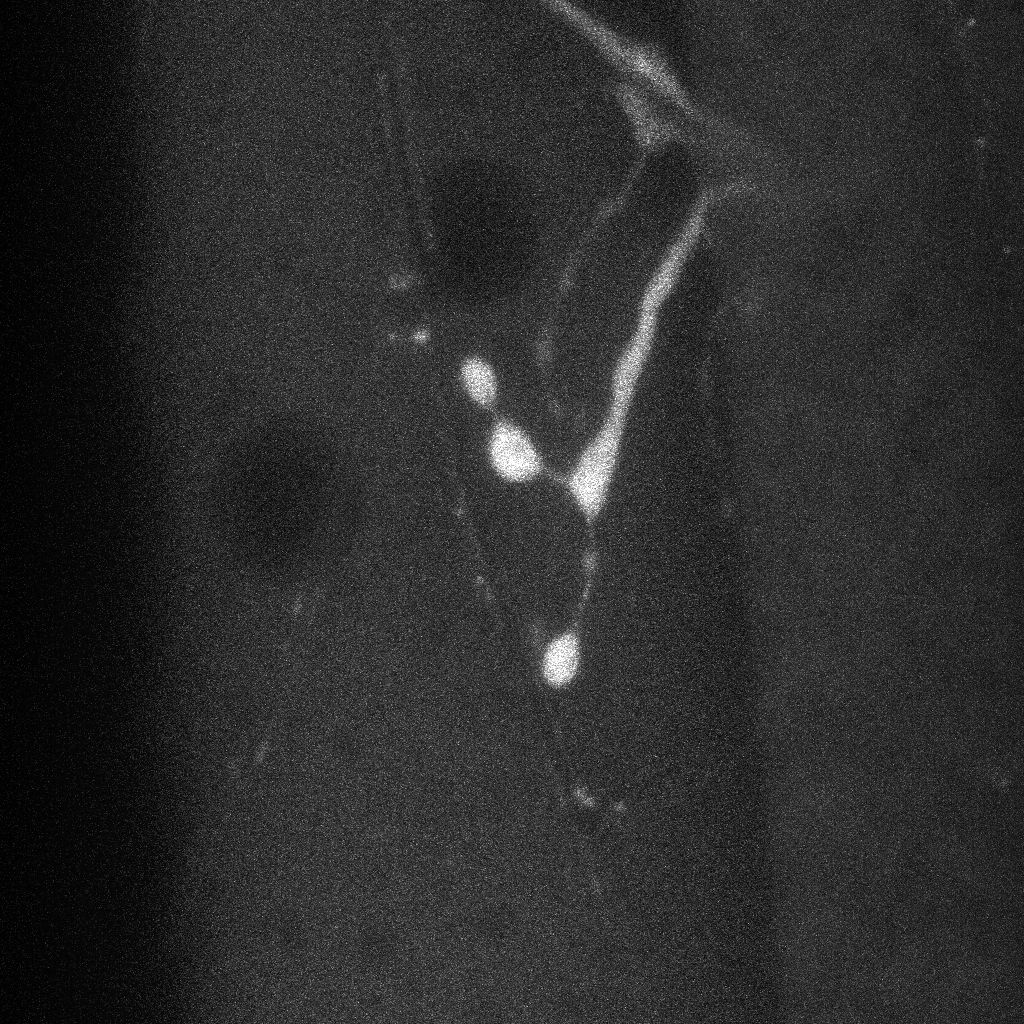

Supplement: Supplementary file 8 — Source data Fig. 3 [file 44318_2025_536_MOESM8_ESM.zip › Figure 3/Figure 3Q/EndoADA.tif]

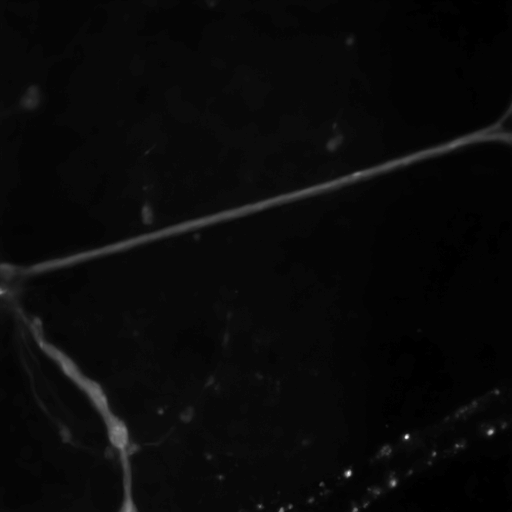

Supplement: Supplementary file 8 — Source data Fig. 3 [file 44318_2025_536_MOESM8_ESM.zip › Figure 3/Figure 3H/rab39KO.tif]

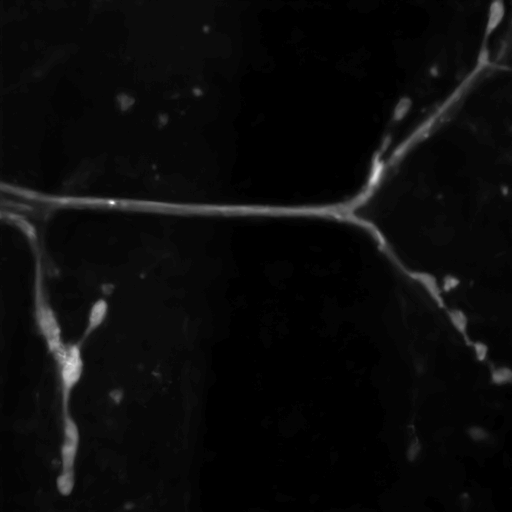

Supplement: Supplementary file 8 — Source data Fig. 3 [file 44318_2025_536_MOESM8_ESM.zip › Figure 3/Figure 3H/w1118.tif]

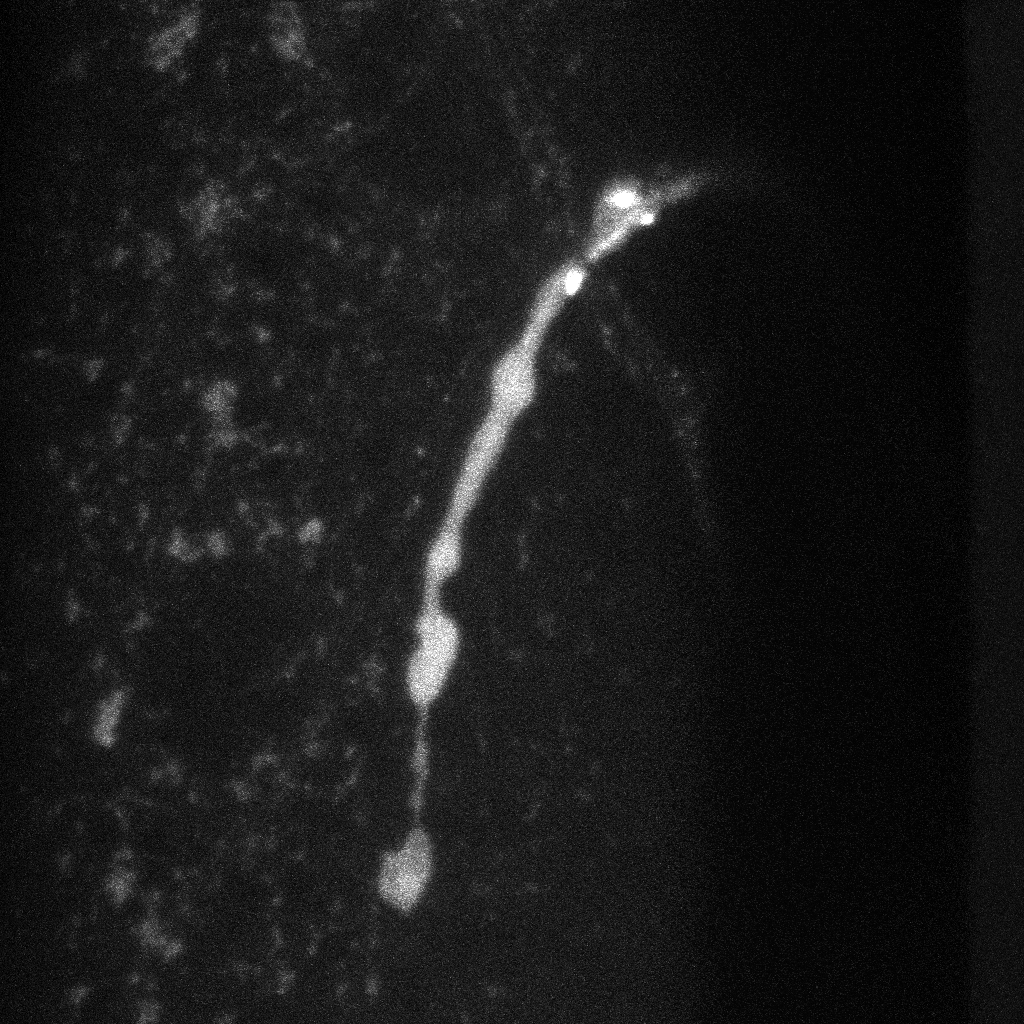

Supplement: Supplementary file 8 — Source data Fig. 3 [file 44318_2025_536_MOESM8_ESM.zip › Figure 3/Figure 3A/rab39KO.tif]

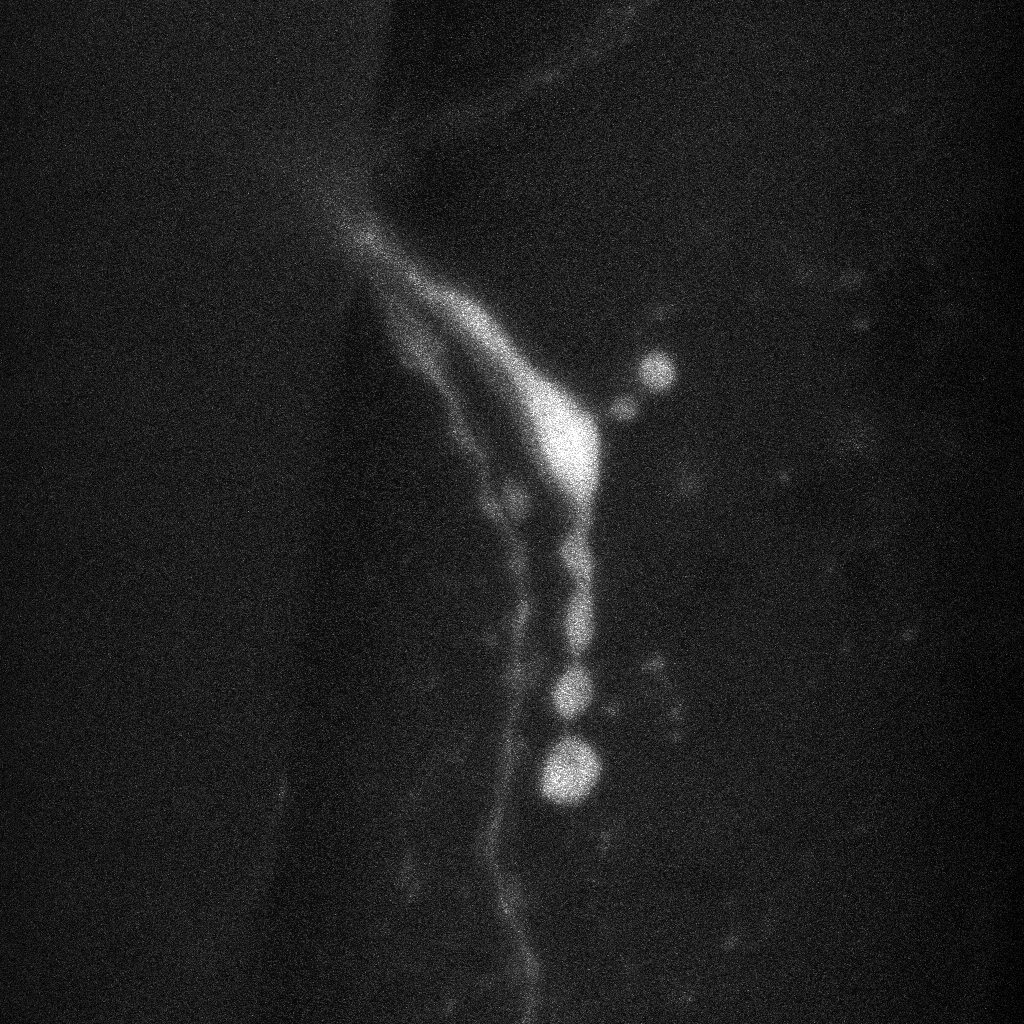

Supplement: Supplementary file 8 — Source data Fig. 3 [file 44318_2025_536_MOESM8_ESM.zip › Figure 3/Figure 3A/w1118.tif]

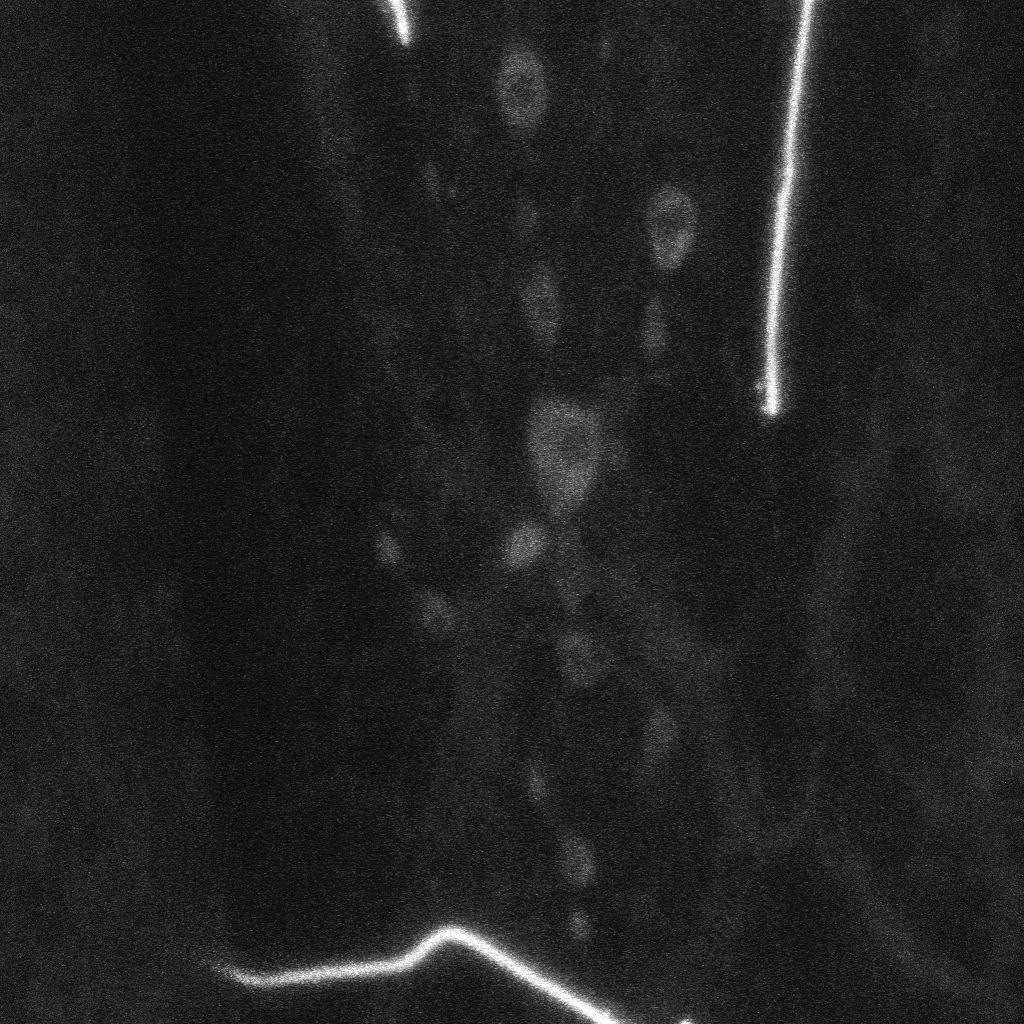

Supplement: Supplementary file 8 — Source data Fig. 3 [file 44318_2025_536_MOESM8_ESM.zip › Figure 3/Figure 3N/rab39KO_57C10_UASnsybslowFT.tif]

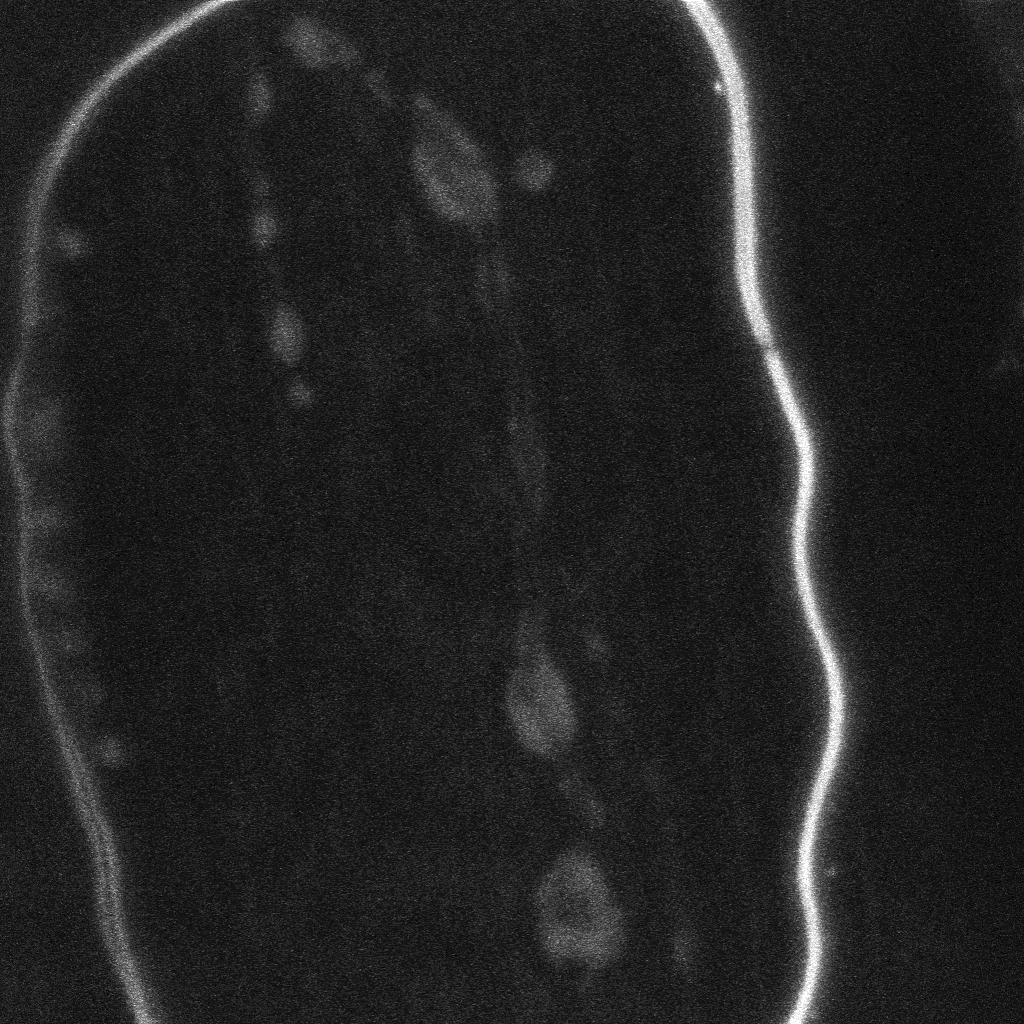

Supplement: Supplementary file 8 — Source data Fig. 3 [file 44318_2025_536_MOESM8_ESM.zip › Figure 3/Figure 3N/w1118_57C10_UASnsybslowFT.tif]

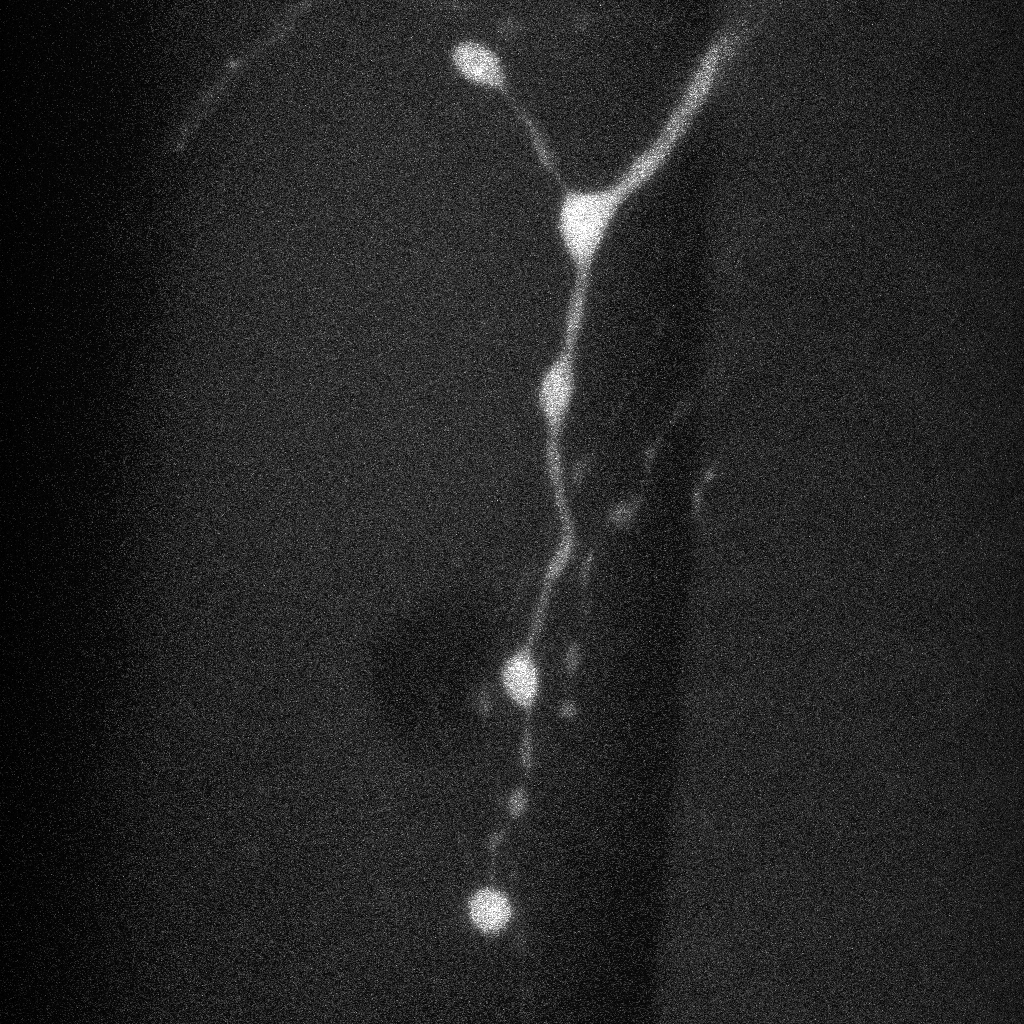

Supplement: Supplementary file 10 — Source data Fig. 5 [file 44318_2025_536_MOESM10_ESM.zip › Figure 5/Figure 5A/w1118.tif]

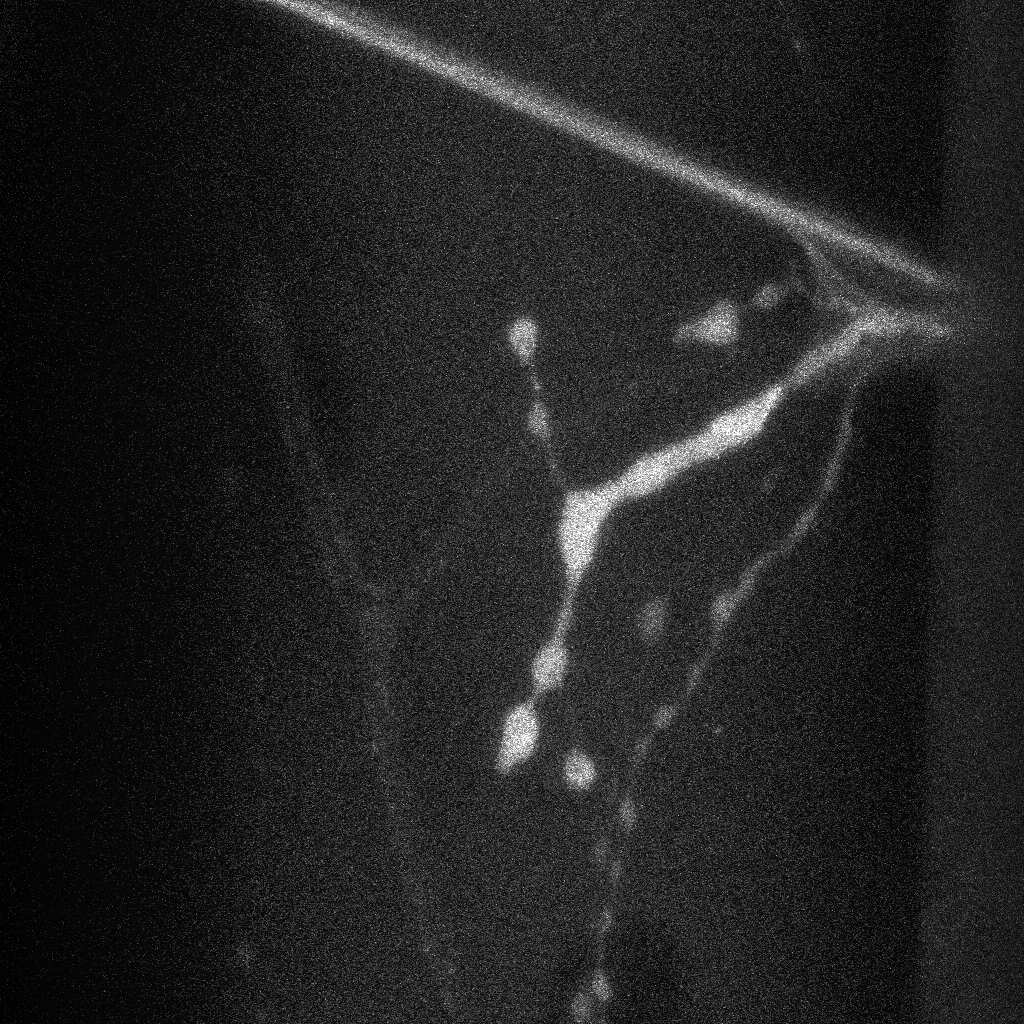

Supplement: Supplementary file 10 — Source data Fig. 5 [file 44318_2025_536_MOESM10_ESM.zip › Figure 5/Figure 5C/rab39KO_UASRab39WT.tif]

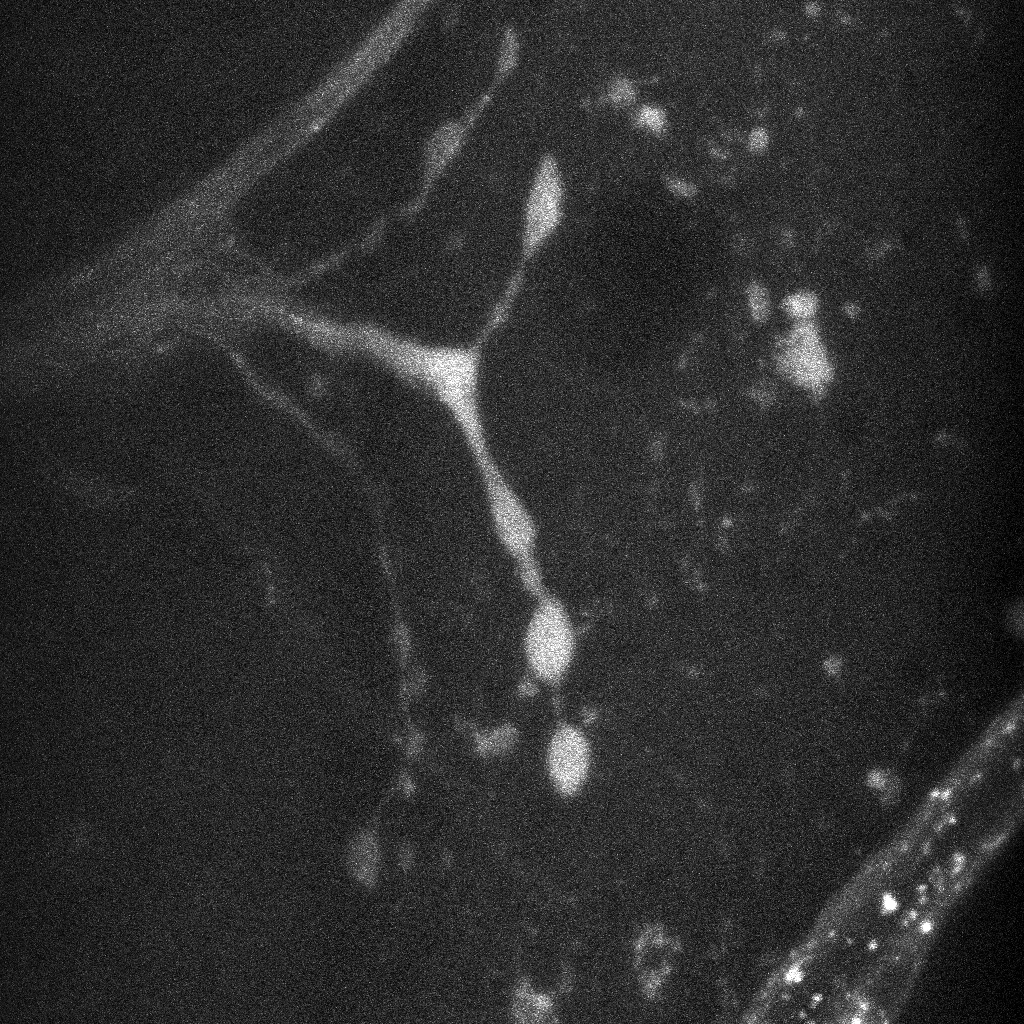

Supplement: Supplementary file 10 — Source data Fig. 5 [file 44318_2025_536_MOESM10_ESM.zip › Figure 5/Figure 5D/rab39KO_UASRAB39CA.tif]

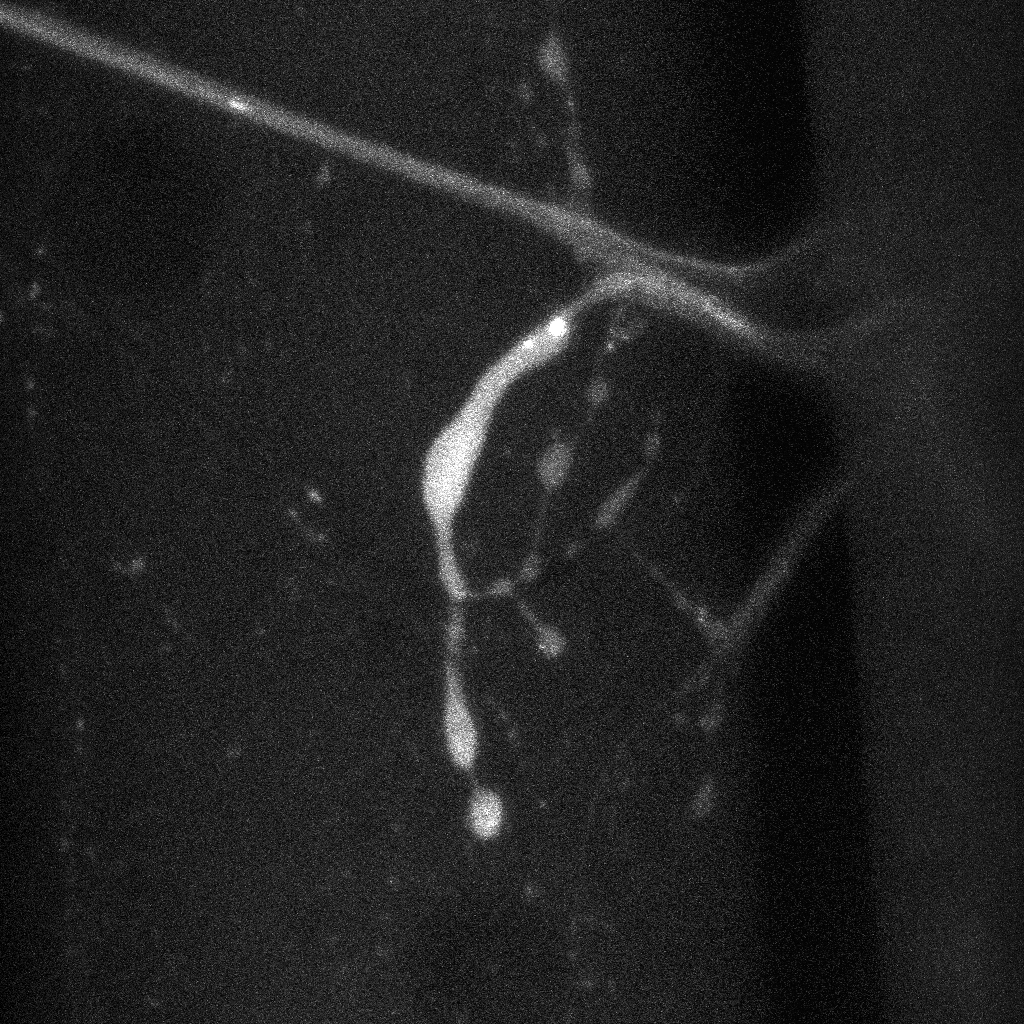

Supplement: Supplementary file 10 — Source data Fig. 5 [file 44318_2025_536_MOESM10_ESM.zip › Figure 5/Figure 5E/rab39KO_UASRab39DN.tif]

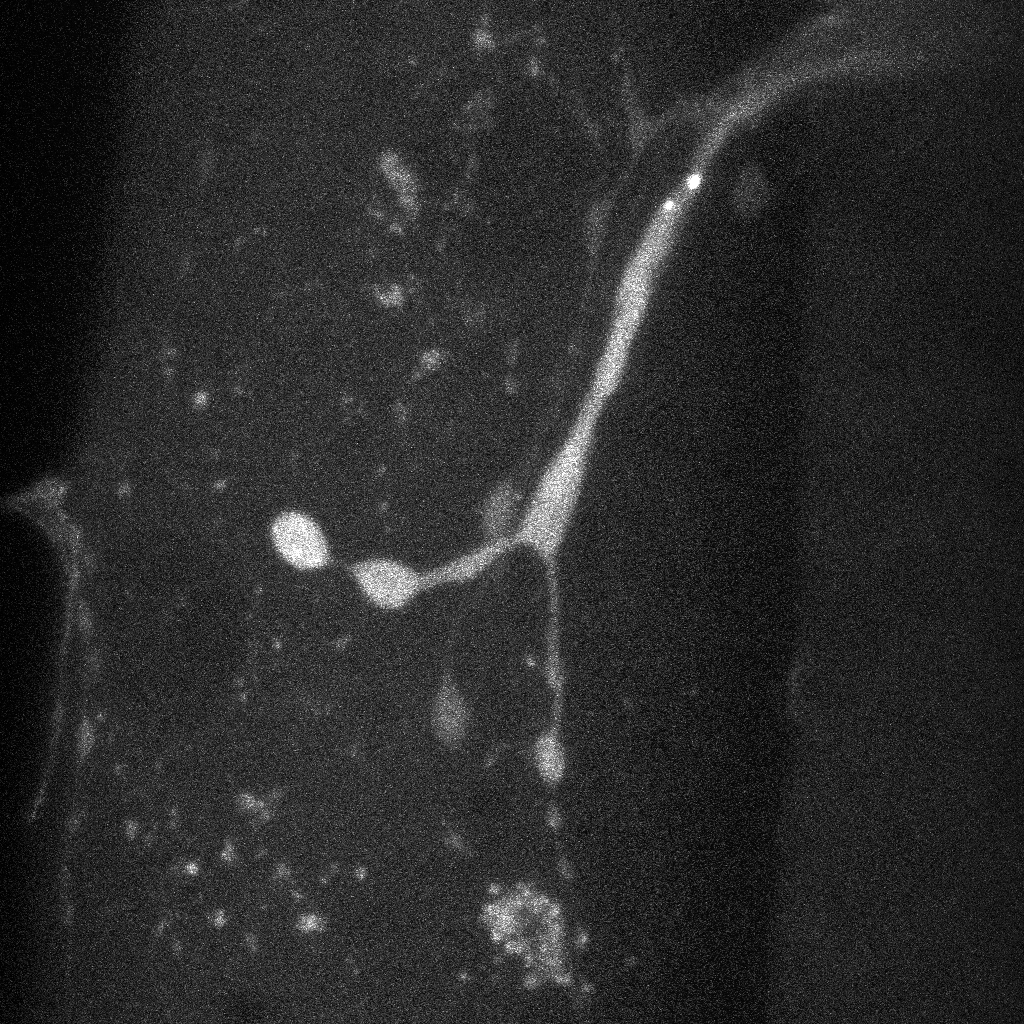

Supplement: Supplementary file 10 — Source data Fig. 5 [file 44318_2025_536_MOESM10_ESM.zip › Figure 5/Figure 5B/rab39KO.tif]

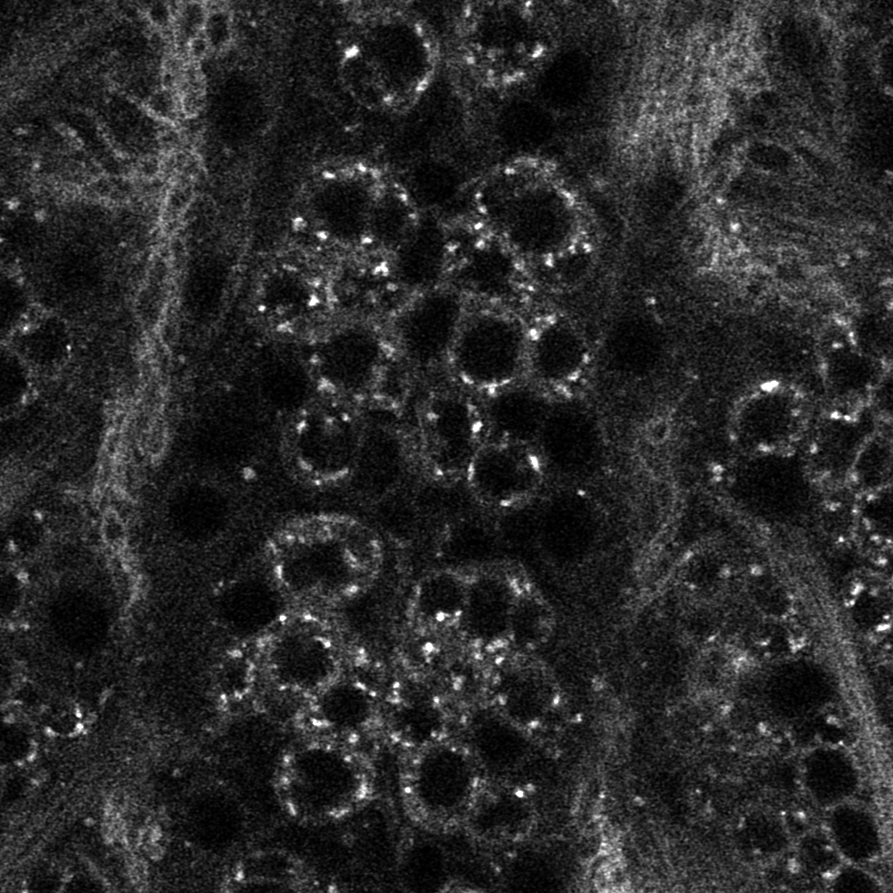

Supplement: Supplementary file 11 — Source data Fig. 6 [file 44318_2025_536_MOESM11_ESM.zip › Figure 6/Figure 6A/eYFP-Rab39.tif]

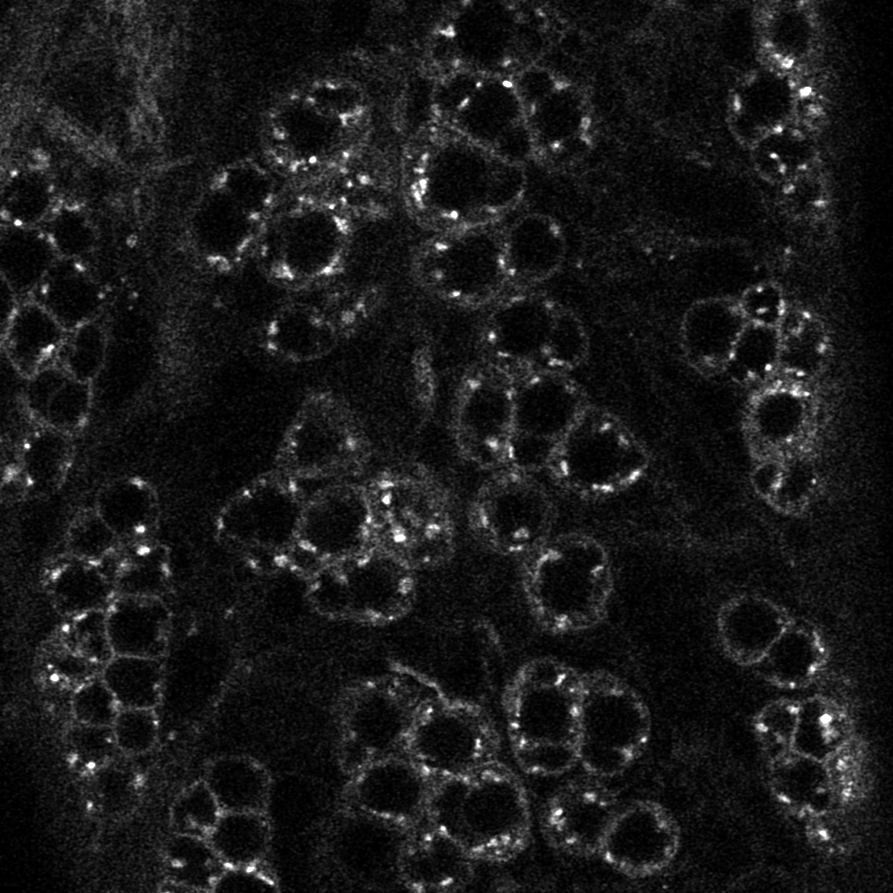

Supplement: Supplementary file 11 — Source data Fig. 6 [file 44318_2025_536_MOESM11_ESM.zip › Figure 6/Figure 6A/w1118.tif]

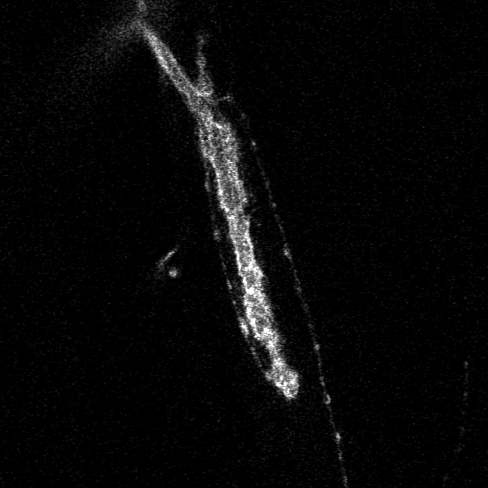

Supplement: Supplementary file 11 — Source data Fig. 6 [file 44318_2025_536_MOESM11_ESM.zip › Figure 6/Figure 6B/w1118.tif]

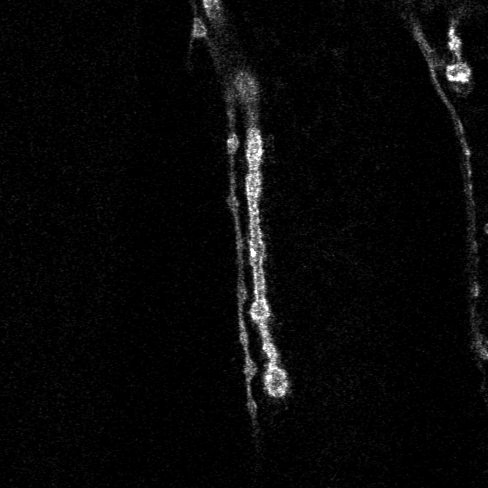

Supplement: Supplementary file 11 — Source data Fig. 6 [file 44318_2025_536_MOESM11_ESM.zip › Figure 6/Figure 6B/eYFPRab39.tif]

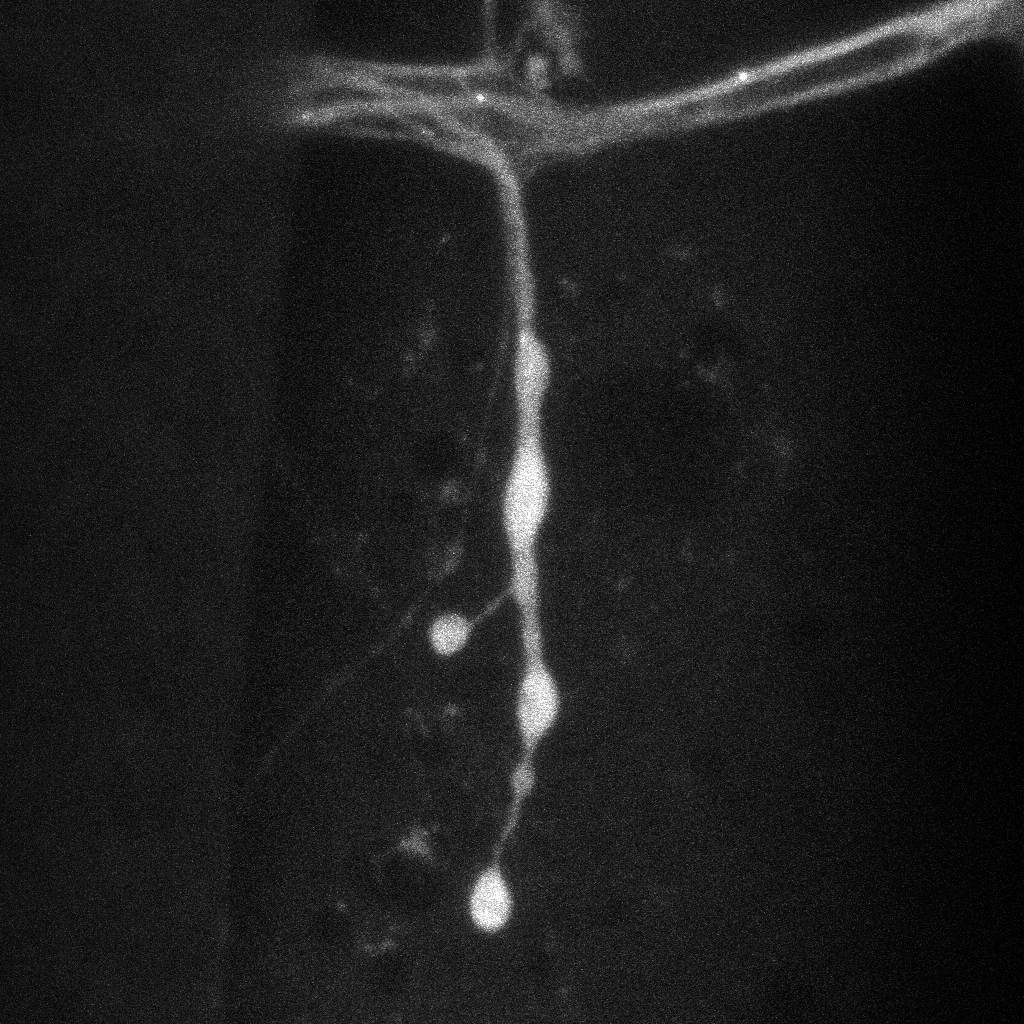

Supplement: Supplementary file 12 — Source data Fig. 7 [file 44318_2025_536_MOESM12_ESM.zip › Figure 7/Figure 7D/rab39KO_BU009.tif]

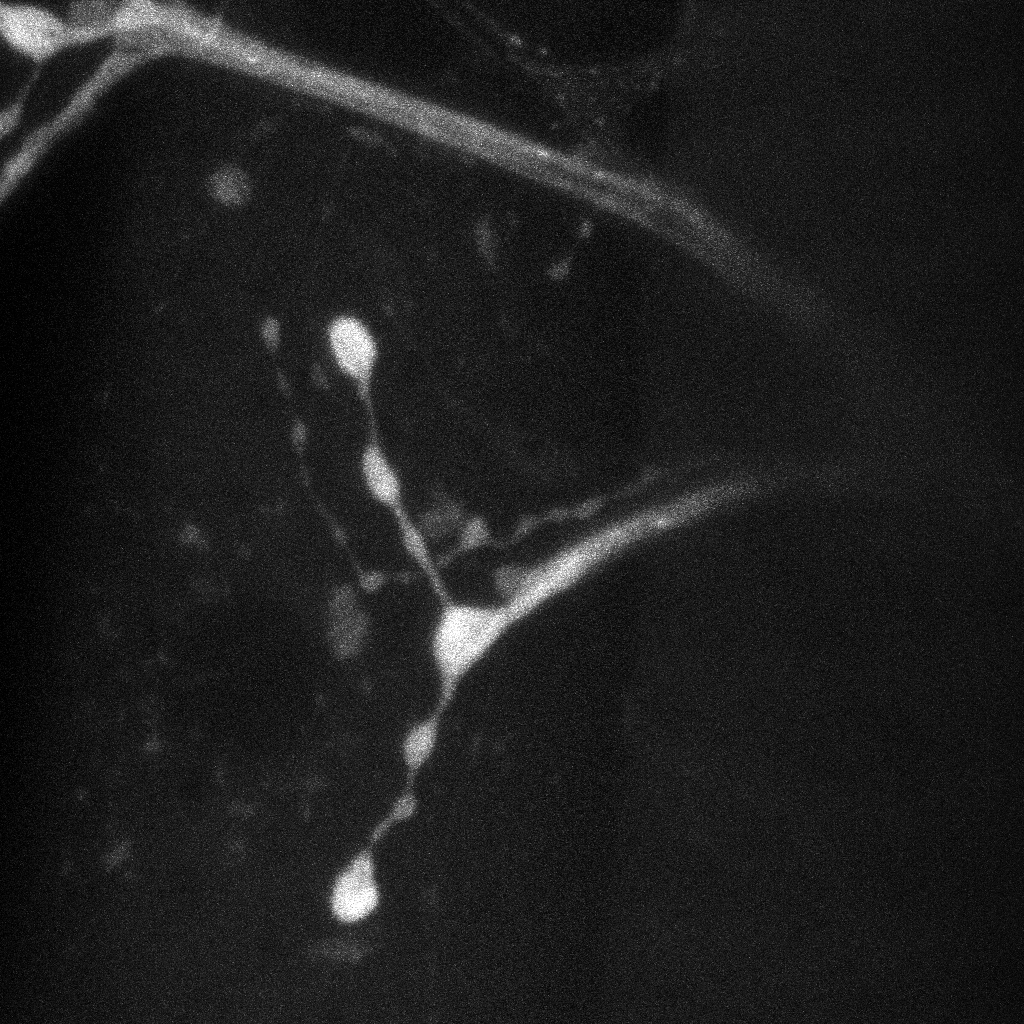

Supplement: Supplementary file 12 — Source data Fig. 7 [file 44318_2025_536_MOESM12_ESM.zip › Figure 7/Figure 7C/rab39KO_shot3.tif]

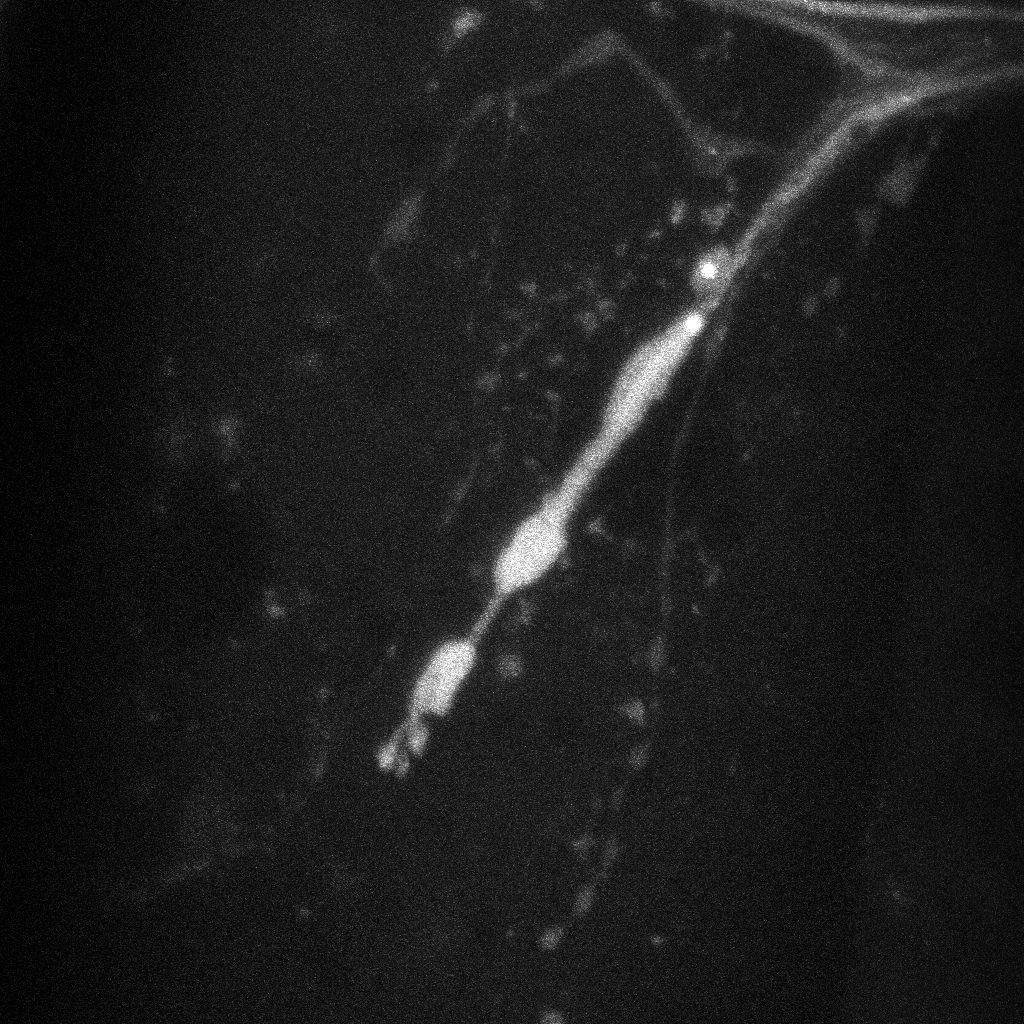

Supplement: Supplementary file 12 — Source data Fig. 7 [file 44318_2025_536_MOESM12_ESM.zip › Figure 7/Figure 7B/rab39KO.tif]

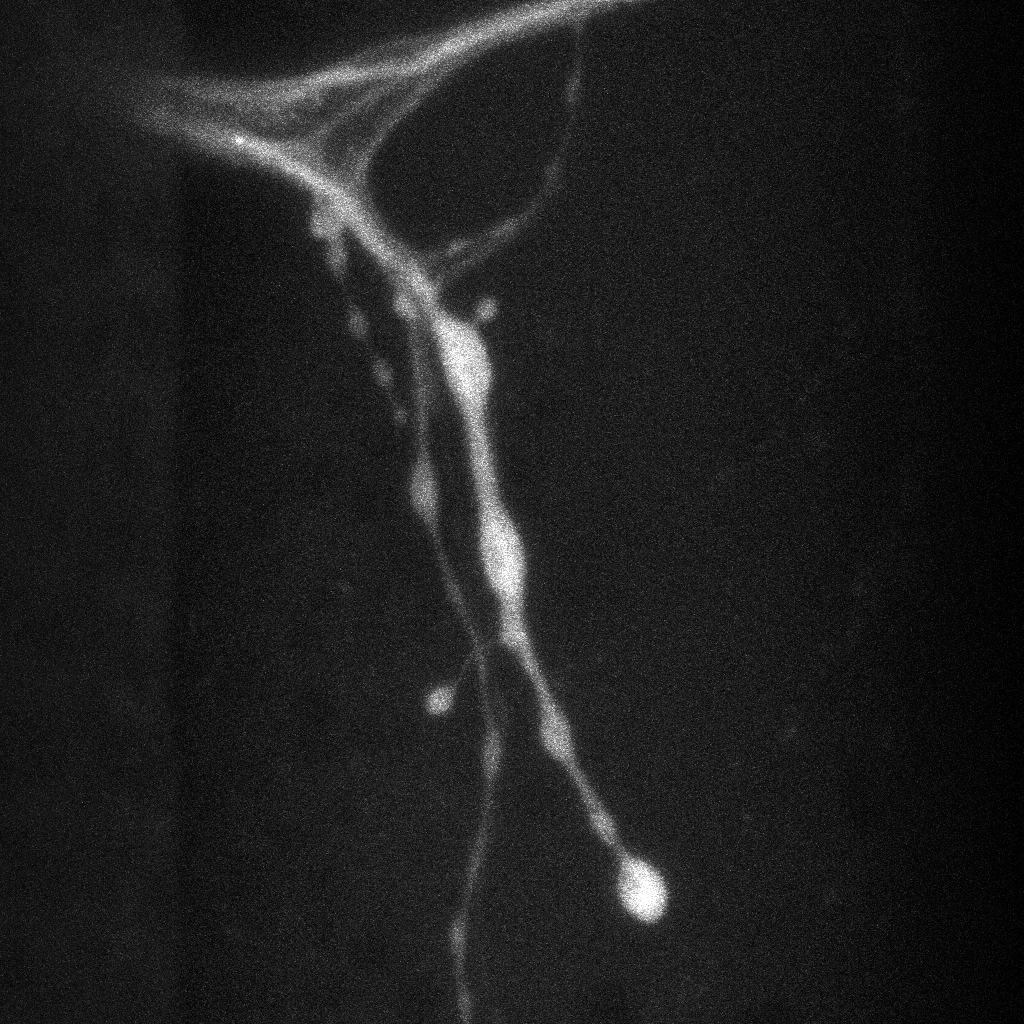

Supplement: Supplementary file 12 — Source data Fig. 7 [file 44318_2025_536_MOESM12_ESM.zip › Figure 7/Figure 7E/rab39KO_R017.tif]

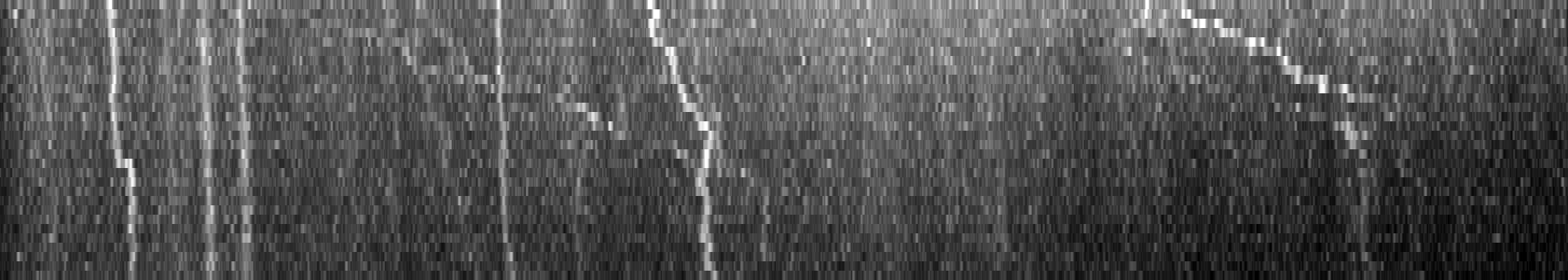

Supplement: Supplementary file 12 — Source data Fig. 7 [file 44318_2025_536_MOESM12_ESM.zip › Figure 7/Figure 7L/rab39KO Projected Kymograph.tif]

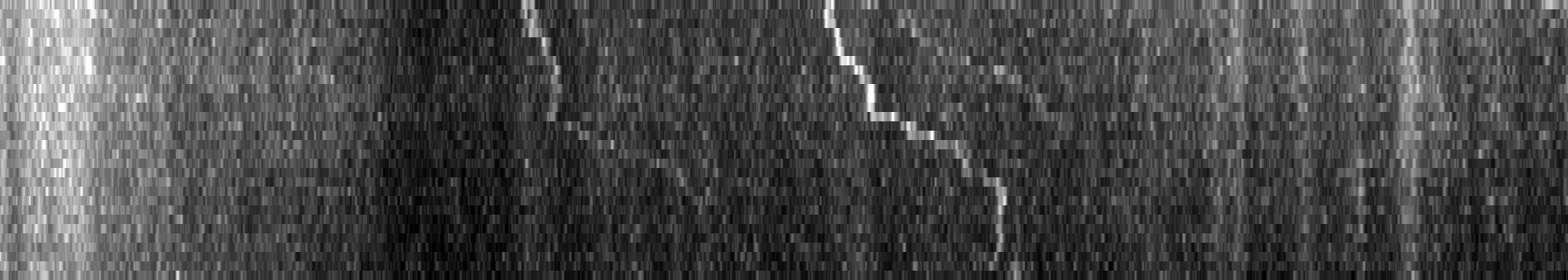

Supplement: Supplementary file 12 — Source data Fig. 7 [file 44318_2025_536_MOESM12_ESM.zip › Figure 7/Figure 7K/CTRL (Projected Kymograph).tif]

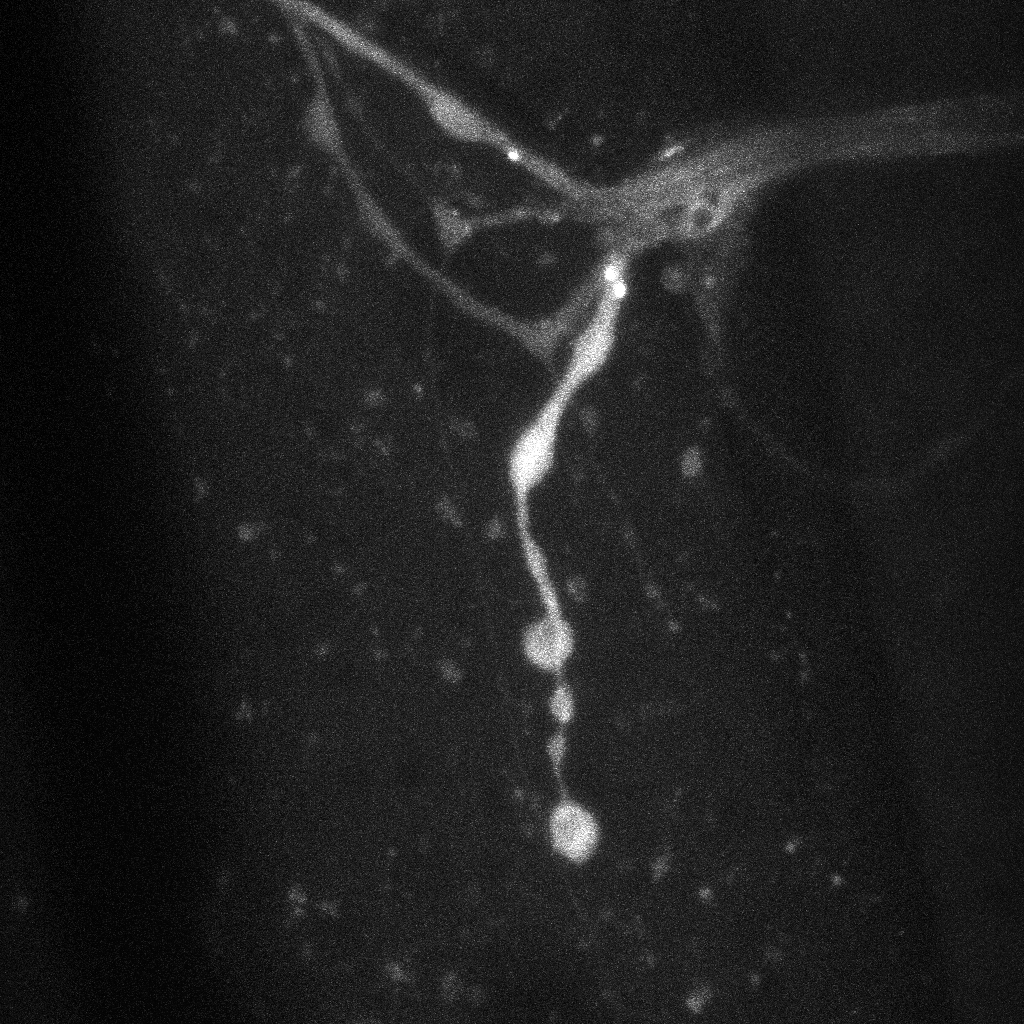

Supplement: Supplementary file 12 — Source data Fig. 7 [file 44318_2025_536_MOESM12_ESM.zip › Figure 7/Figure 7F/rab39KO_shot3_shot+.tif]

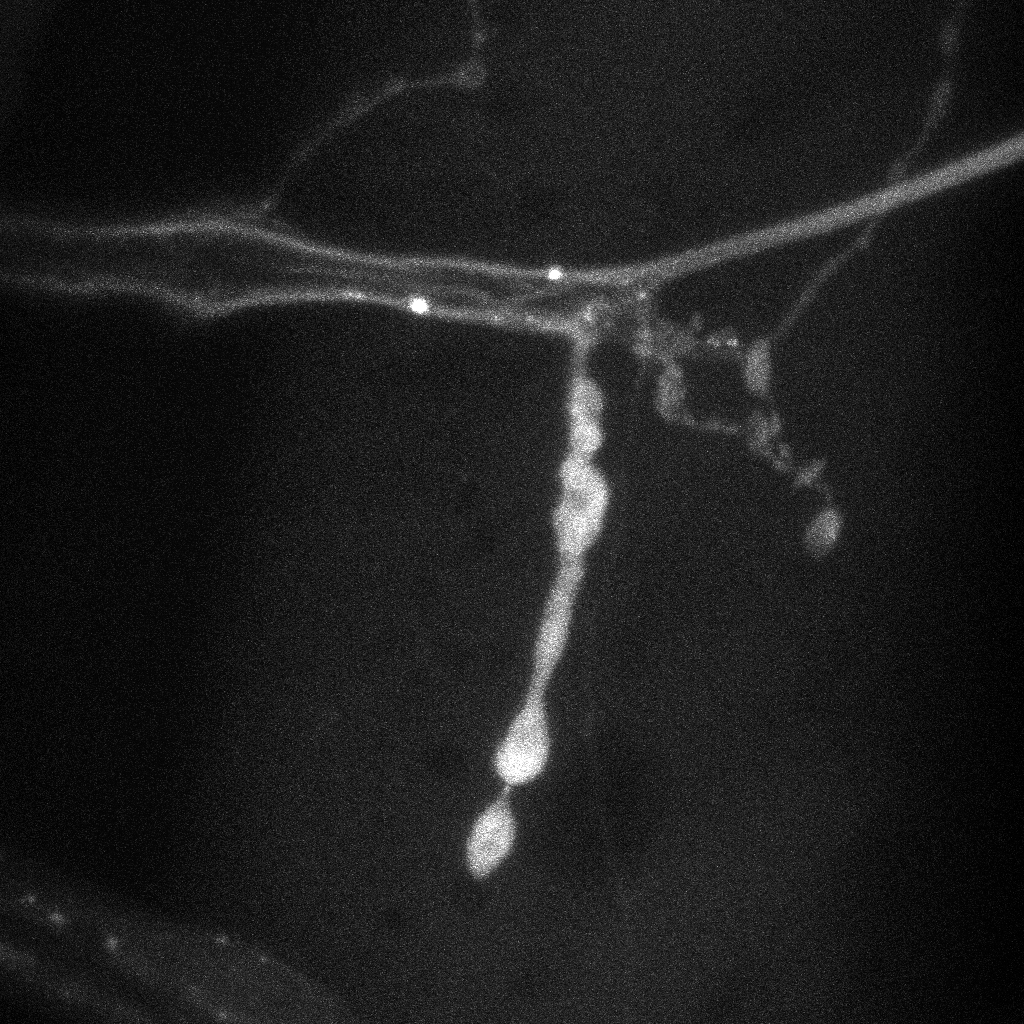

Supplement: Supplementary file 12 — Source data Fig. 7 [file 44318_2025_536_MOESM12_ESM.zip › Figure 7/Figure 7A/w1118.tif]

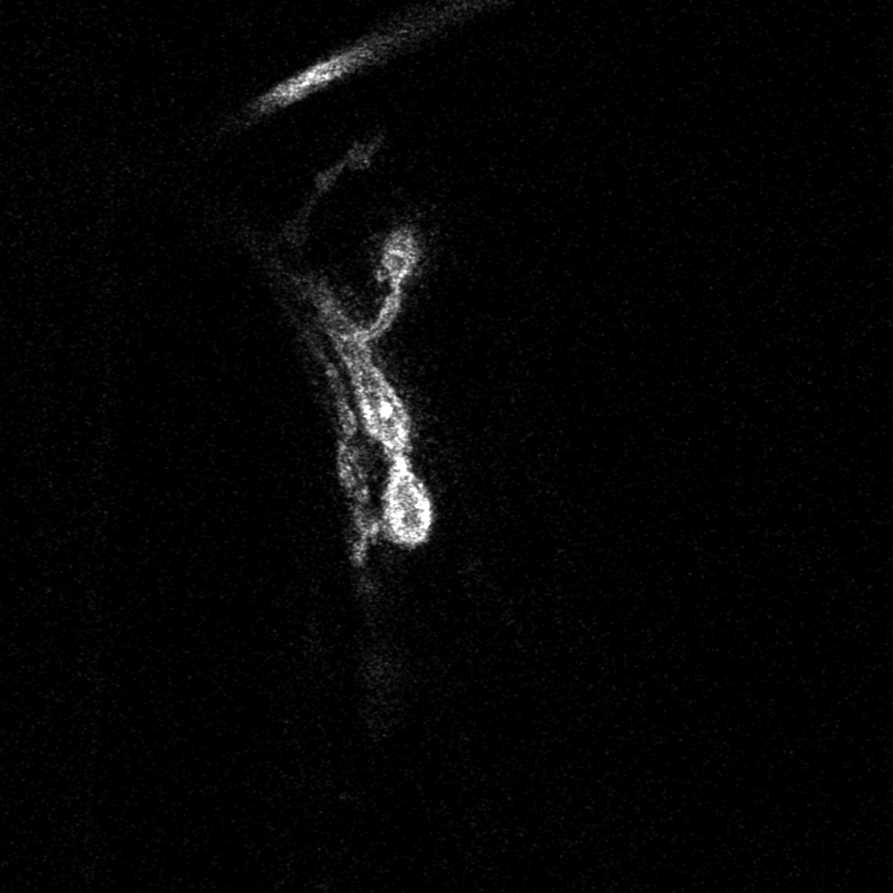

Supplement: Supplementary file 13 — Source data Fig. 8 [file 44318_2025_536_MOESM13_ESM.zip › Figure 8/Figure 8A/rab39KO.tif]

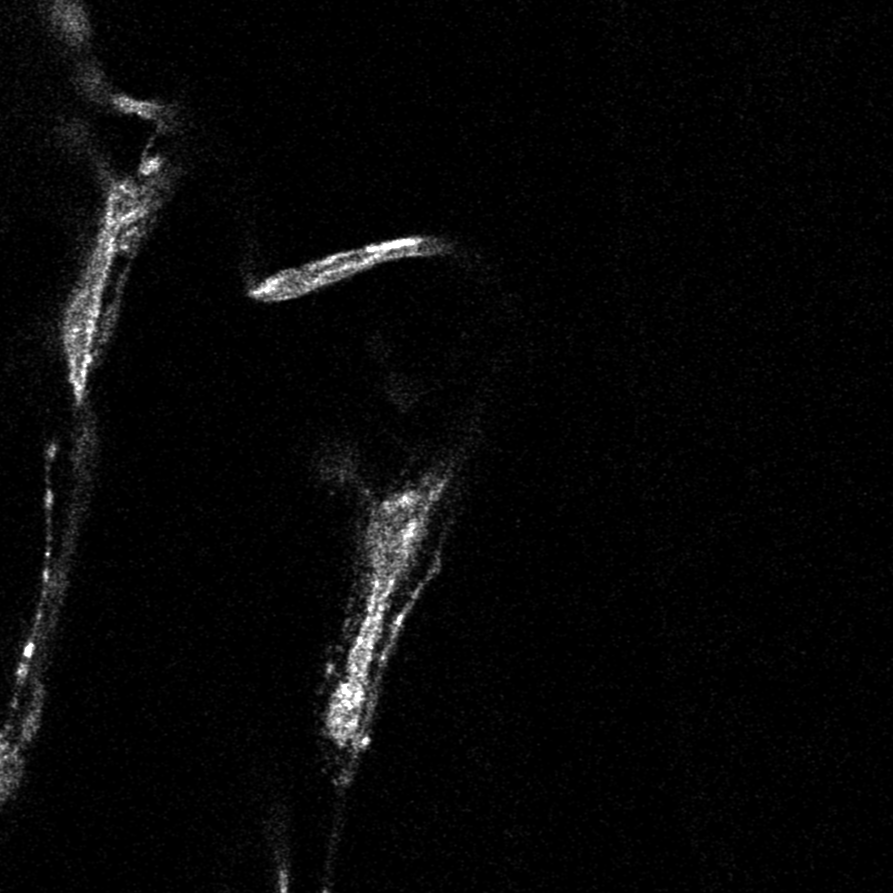

Supplement: Supplementary file 13 — Source data Fig. 8 [file 44318_2025_536_MOESM13_ESM.zip › Figure 8/Figure 8A/CTRL.tif]

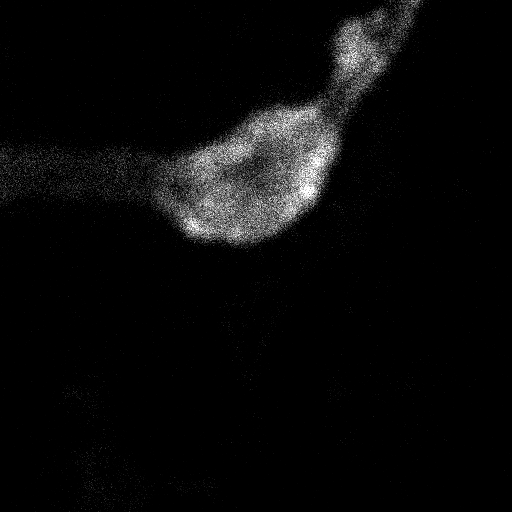

Supplement: Supplementary file 13 — Source data Fig. 8 [file 44318_2025_536_MOESM13_ESM.zip › Figure 8/Figure 8C/rab39KO.tif]

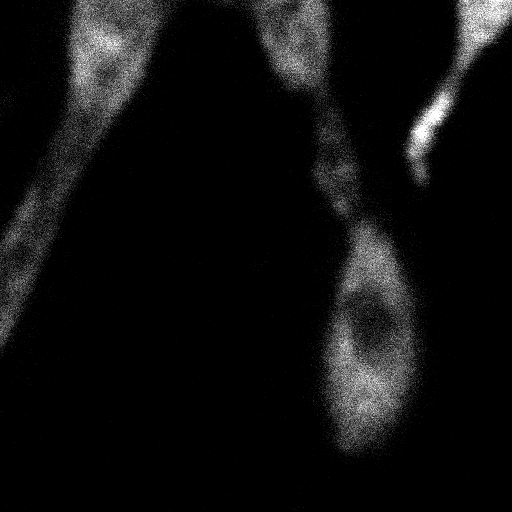

Supplement: Supplementary file 13 — Source data Fig. 8 [file 44318_2025_536_MOESM13_ESM.zip › Figure 8/Figure 8C/CTRL.tif]

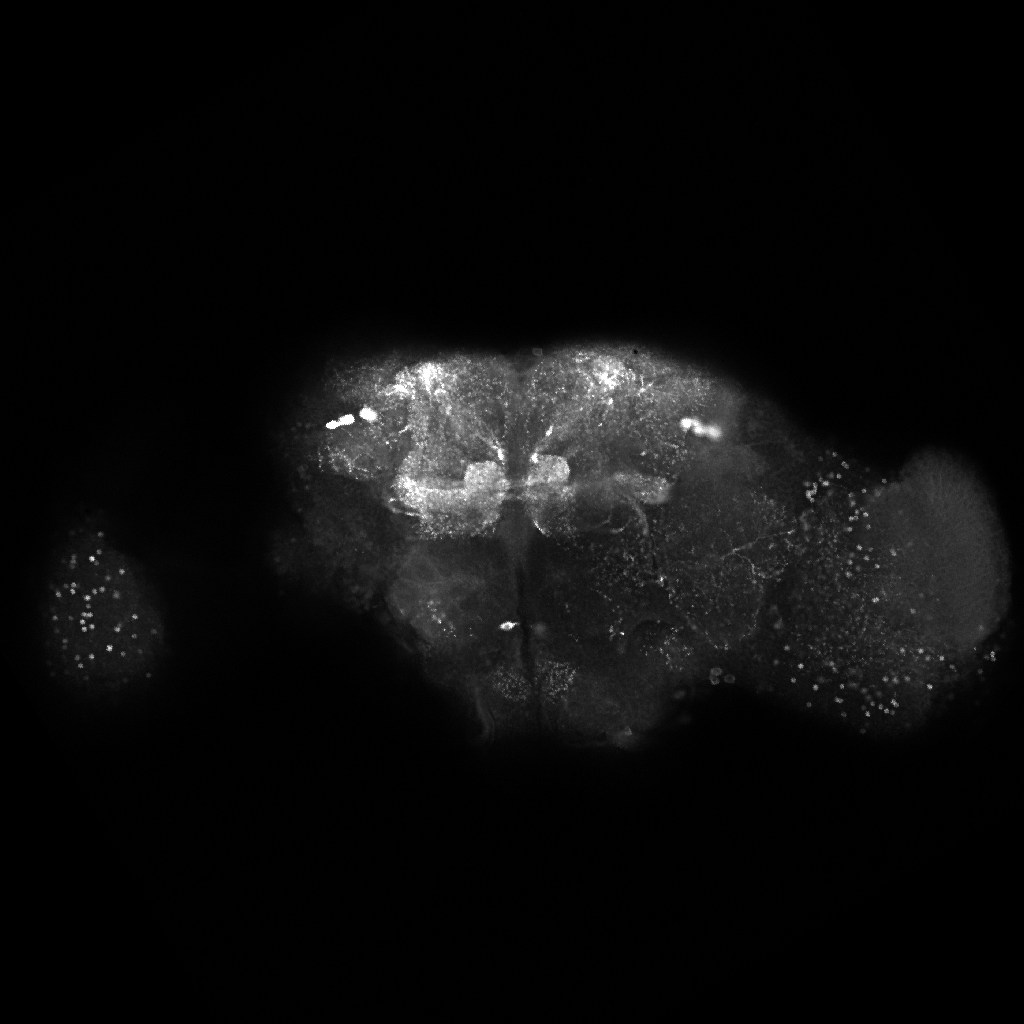

Supplement: Supplementary file 14 — Source data Fig. 9 [file 44318_2025_536_MOESM14_ESM.zip › Figure 9/Figure 9C_C'/rab39KO.tif]

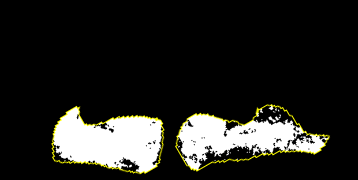

Supplement: Supplementary file 14 — Source data Fig. 9 [file 44318_2025_536_MOESM14_ESM.zip › Figure 9/Figure 9C_C'/rab39KO_masked.tif]

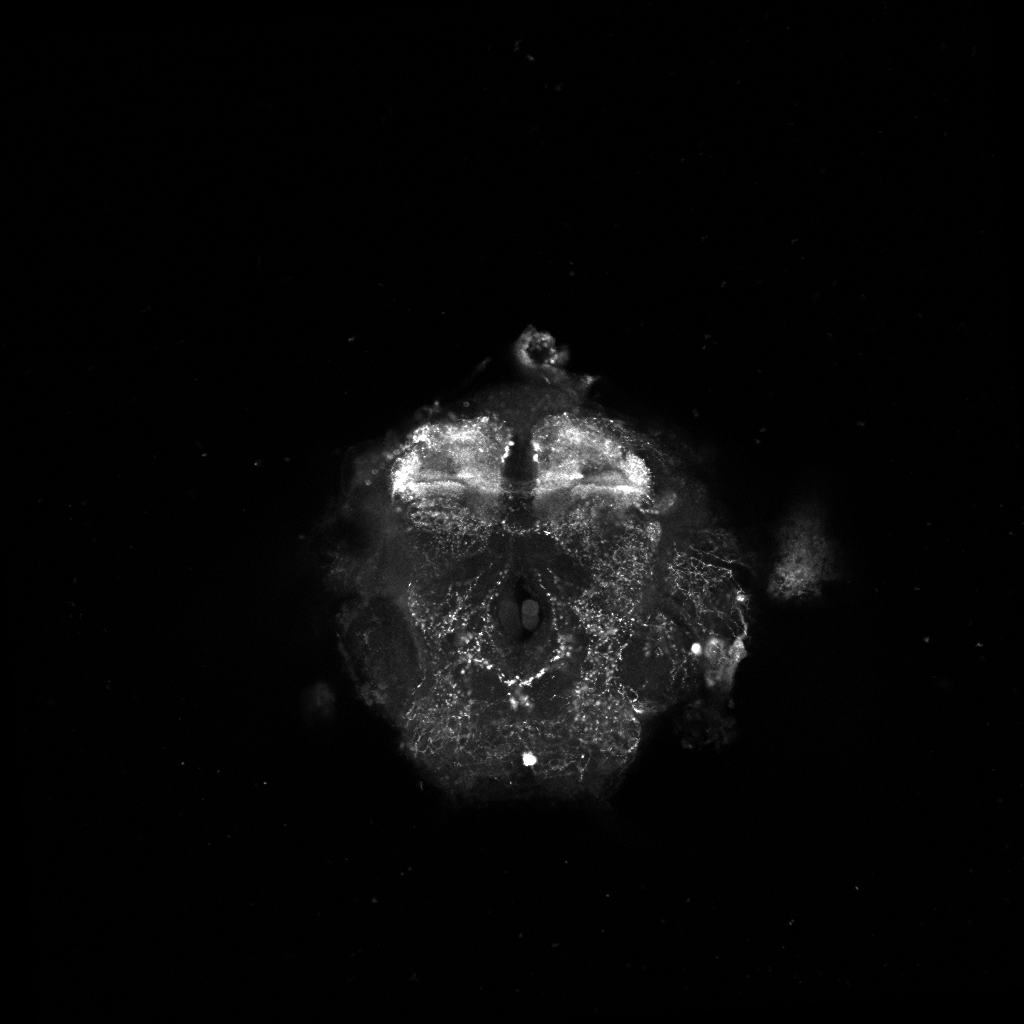

Supplement: Supplementary file 14 — Source data Fig. 9 [file 44318_2025_536_MOESM14_ESM.zip › Figure 9/Figure 9G_G'/rab39KO.tif]

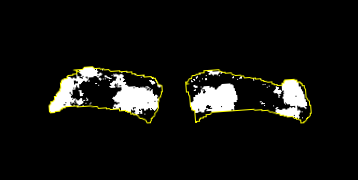

Supplement: Supplementary file 14 — Source data Fig. 9 [file 44318_2025_536_MOESM14_ESM.zip › Figure 9/Figure 9G_G'/rab39KO_masked.tif]

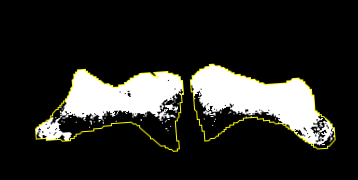

Supplement: Supplementary file 14 — Source data Fig. 9 [file 44318_2025_536_MOESM14_ESM.zip › Figure 9/Figure 9E_E'/rab39KO_shot3:+_masked.tif]

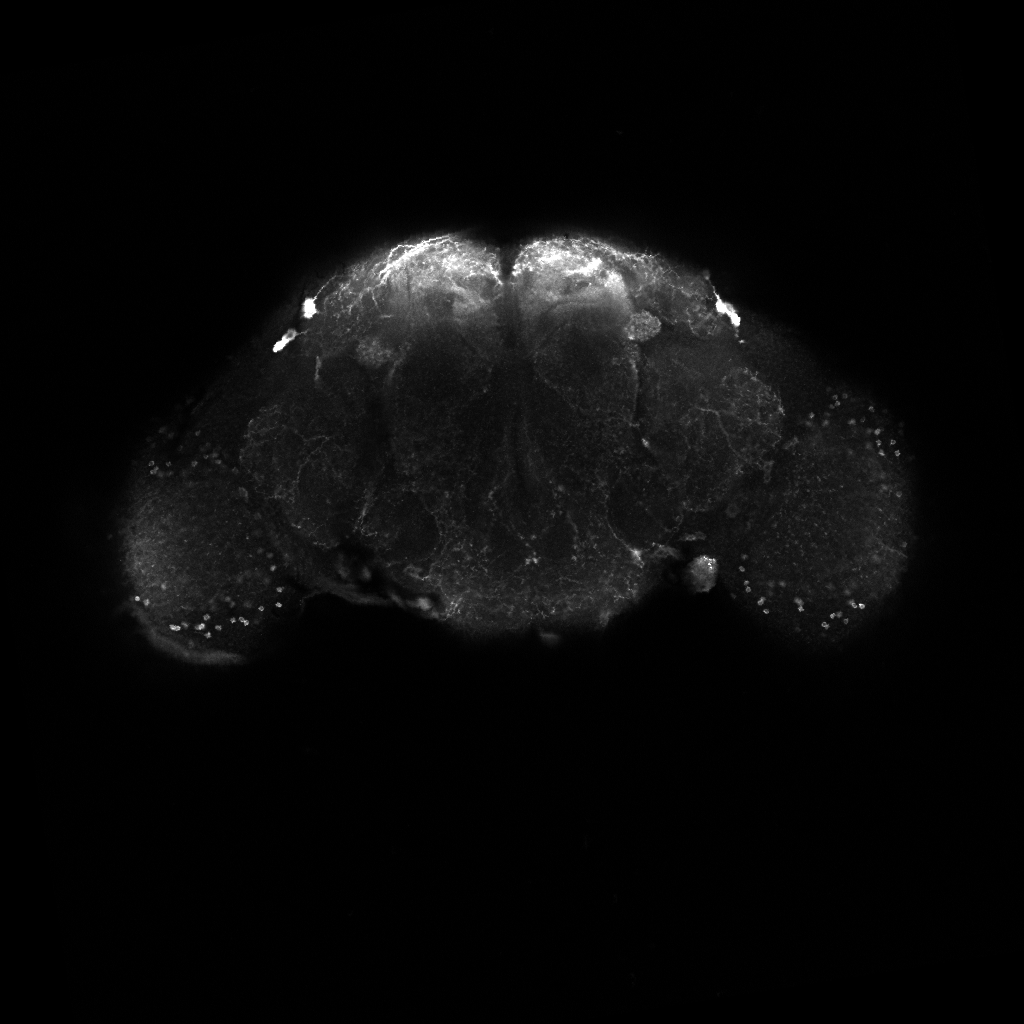

Supplement: Supplementary file 14 — Source data Fig. 9 [file 44318_2025_536_MOESM14_ESM.zip › Figure 9/Figure 9E_E'/rab39KO_shot3:+.tif]

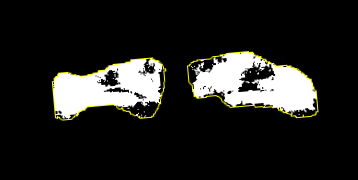

Supplement: Supplementary file 14 — Source data Fig. 9 [file 44318_2025_536_MOESM14_ESM.zip › Figure 9/Figure 9I_I'/rab39KO_shot3:+_masked.tif]

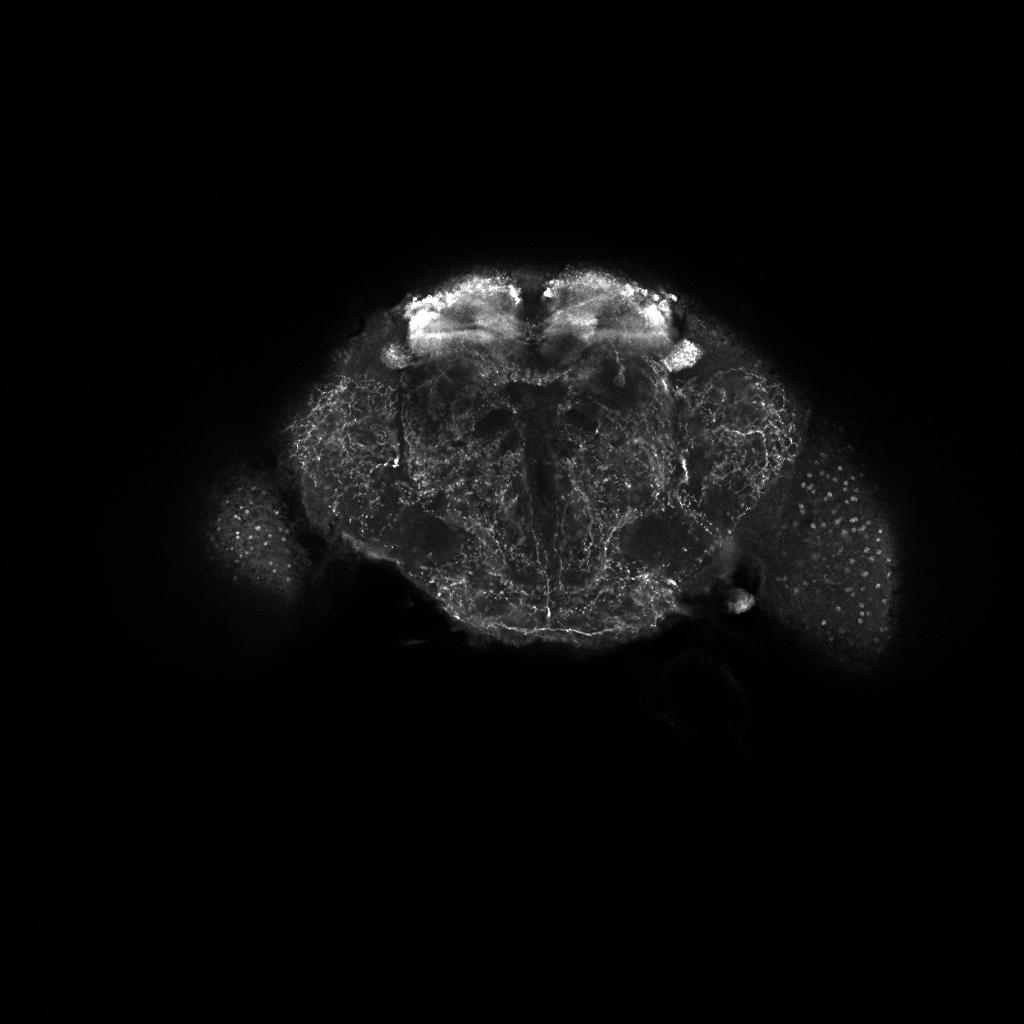

Supplement: Supplementary file 14 — Source data Fig. 9 [file 44318_2025_536_MOESM14_ESM.zip › Figure 9/Figure 9I_I'/rab39KO_shot3:+.tif]

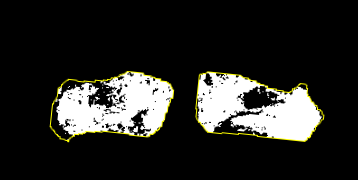

Supplement: Supplementary file 14 — Source data Fig. 9 [file 44318_2025_536_MOESM14_ESM.zip › Figure 9/Figure 9F_F'/CTRL_masked.tif]

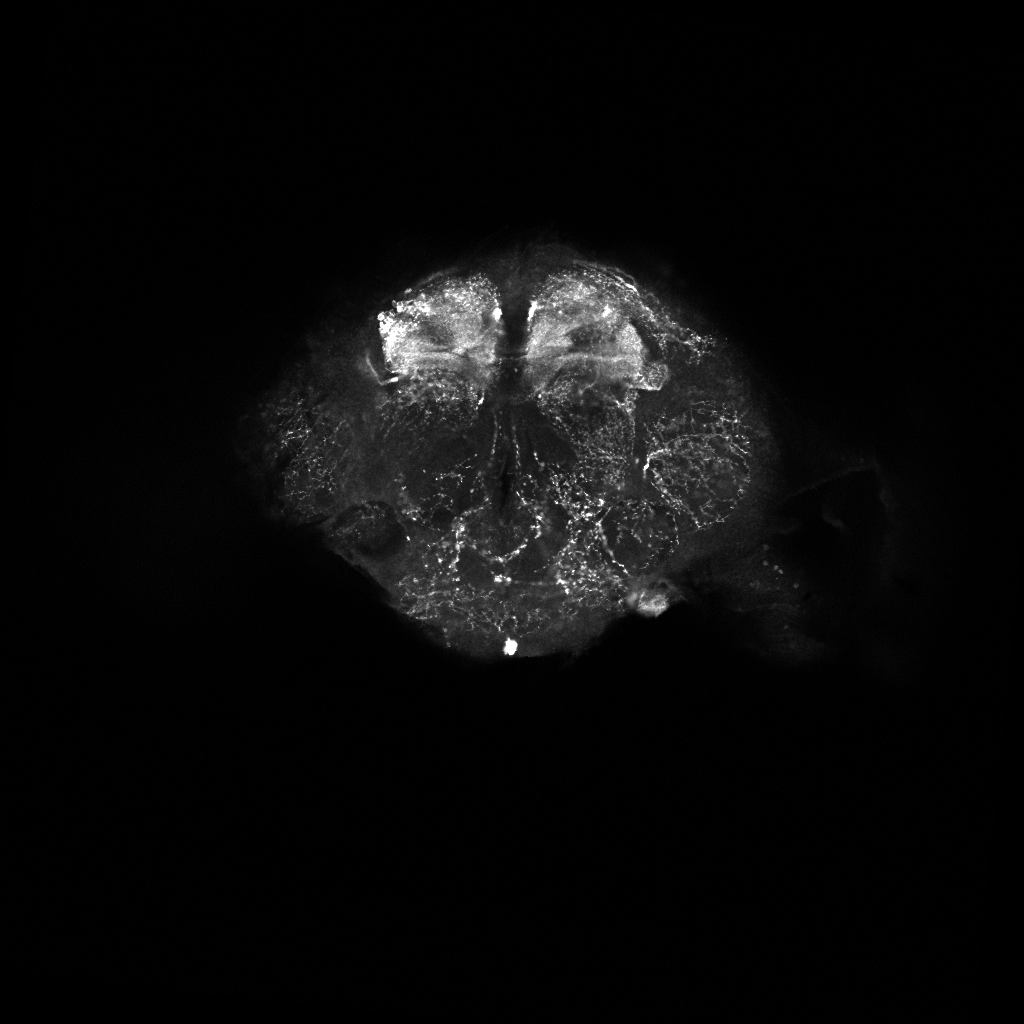

Supplement: Supplementary file 14 — Source data Fig. 9 [file 44318_2025_536_MOESM14_ESM.zip › Figure 9/Figure 9F_F'/CTRL.tif]

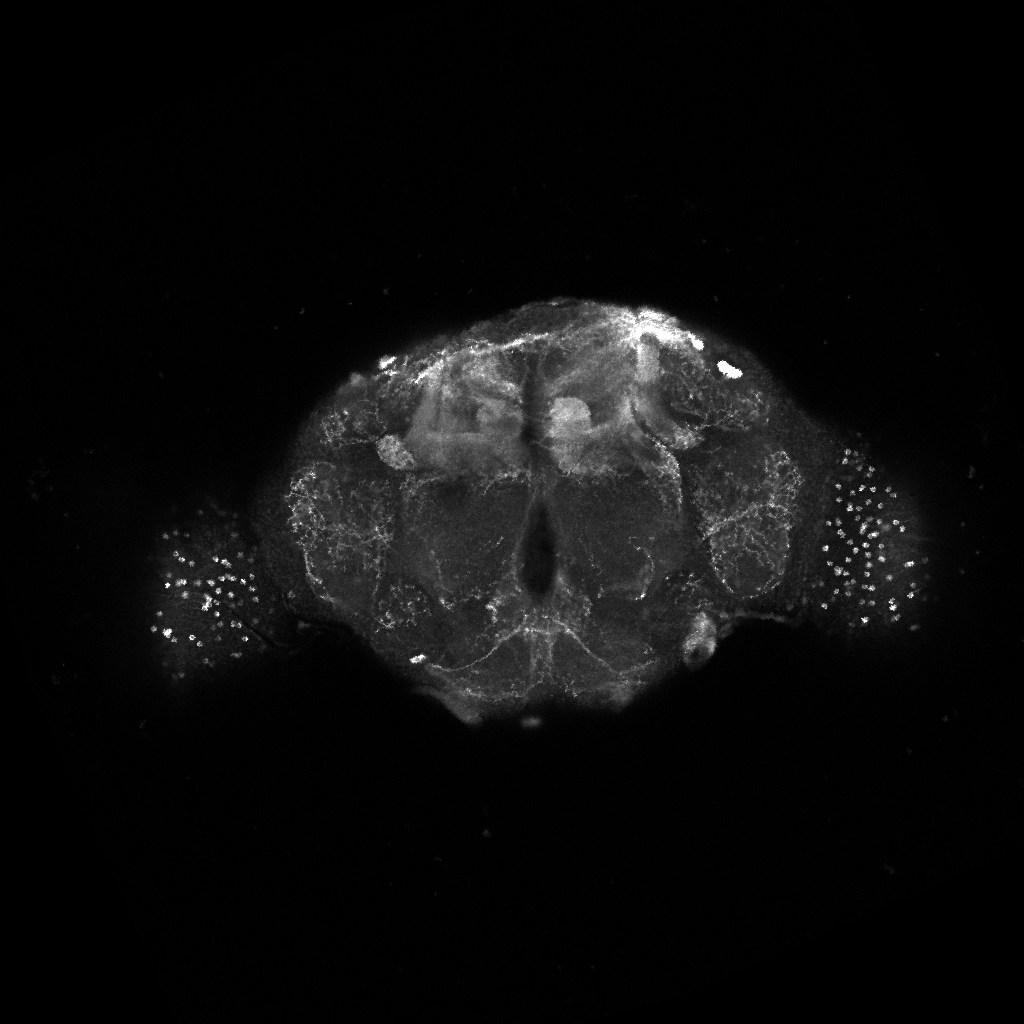

Supplement: Supplementary file 14 — Source data Fig. 9 [file 44318_2025_536_MOESM14_ESM.zip › Figure 9/Figure 9D_D'/shot3:+.tif]

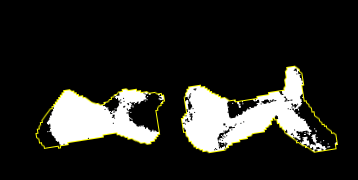

Supplement: Supplementary file 14 — Source data Fig. 9 [file 44318_2025_536_MOESM14_ESM.zip › Figure 9/Figure 9D_D'/shot3:+_masked.tif]

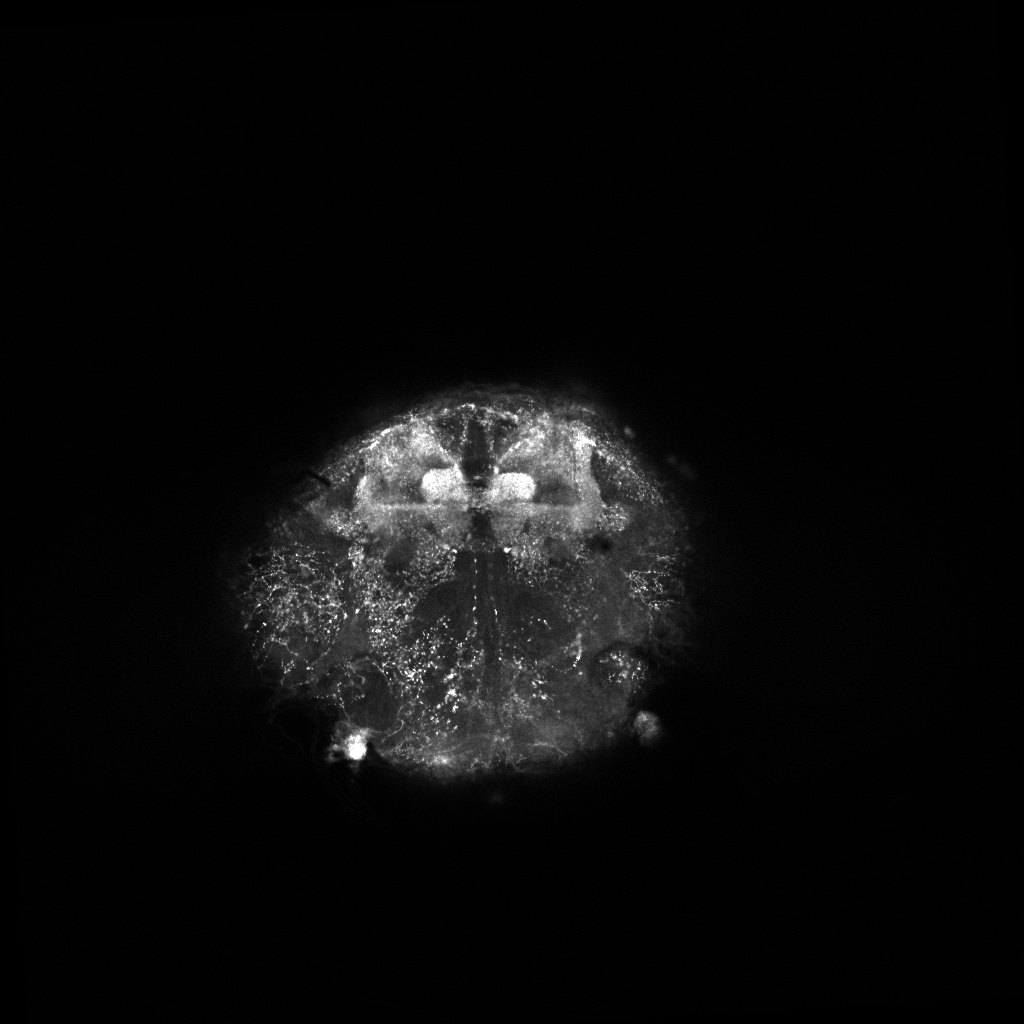

Supplement: Supplementary file 14 — Source data Fig. 9 [file 44318_2025_536_MOESM14_ESM.zip › Figure 9/Figure 9H_H'/shot3:+.tif]

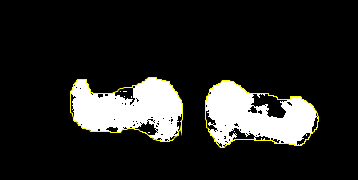

Supplement: Supplementary file 14 — Source data Fig. 9 [file 44318_2025_536_MOESM14_ESM.zip › Figure 9/Figure 9H_H'/shot3:+_masked.tif]

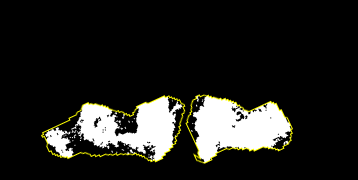

Supplement: Supplementary file 14 — Source data Fig. 9 [file 44318_2025_536_MOESM14_ESM.zip › Figure 9/Figure 9B_B'/CTRL_masked.tif]

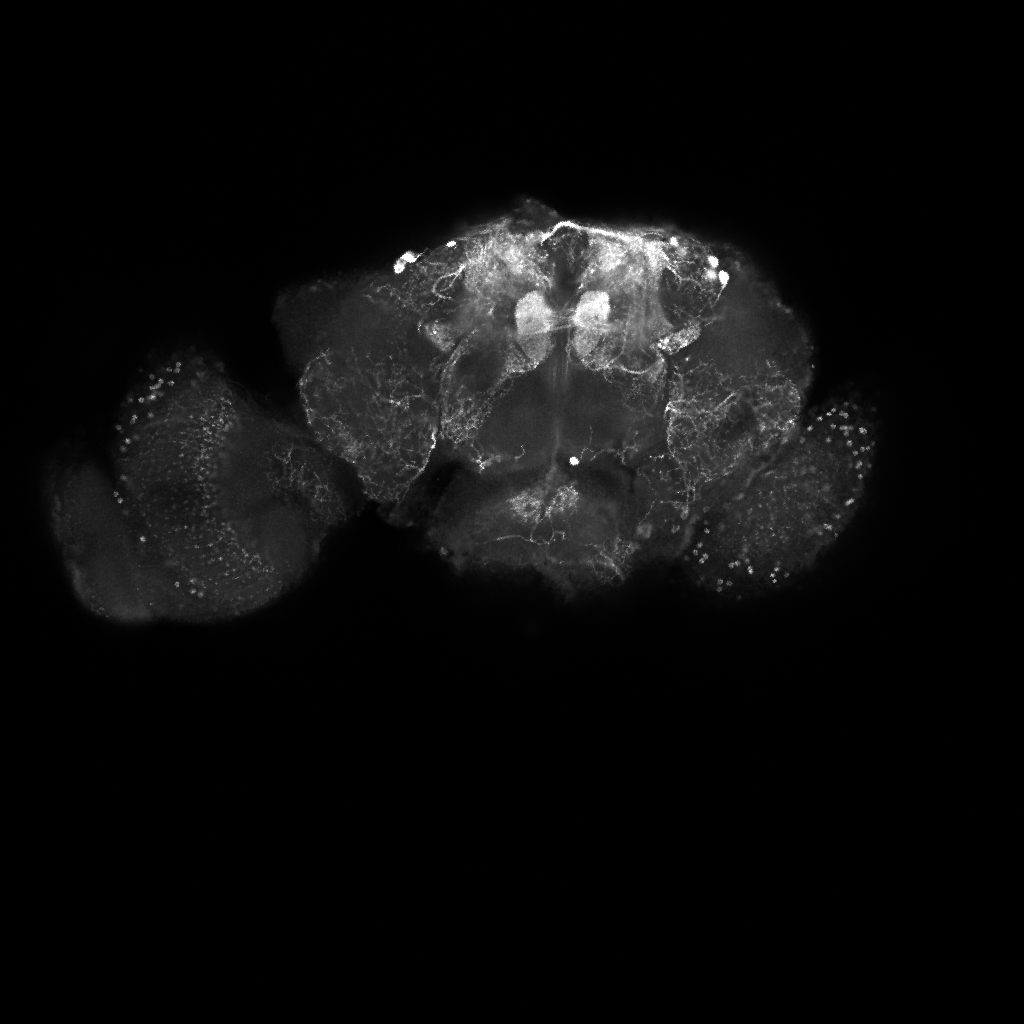

Supplement: Supplementary file 14 — Source data Fig. 9 [file 44318_2025_536_MOESM14_ESM.zip › Figure 9/Figure 9B_B'/CTRL.tif]
