# Supplementary material for: Soma-localized Rab39 inhibits synaptic autophagy by controlling trafficking of Atg9 vesicles
Source: EMBO J. 2025 Aug 21;44(20):5662–93. doi: 10.1038/s44318-025-00536-8 (PMC12528412; doi:10.1038/s44318-025-00536-8)
Supplement: Supplementary file 15 — Expanded View Figures [file 44318_2025_536_MOESM15_ESM.pdf]

Expanded View Figures

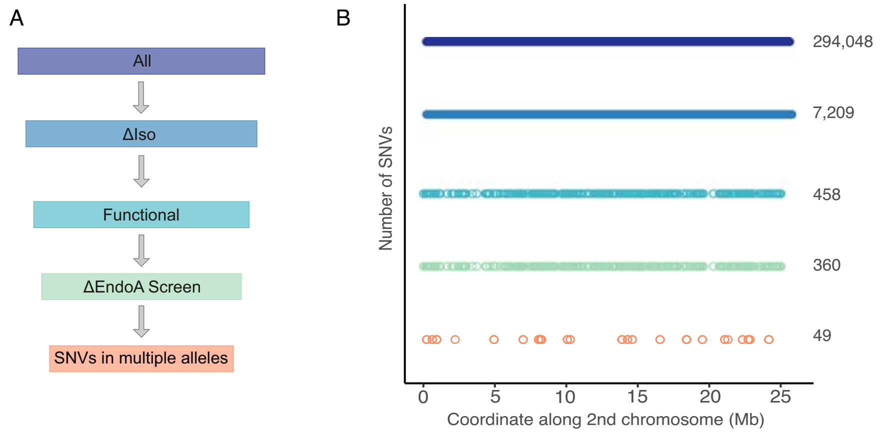

Figure EV1. Filtering process to identify candidate mutations in heterozygous EMS lines.

(**A**) Flowchart summarizing the filtering pipeline used to identify candidate mutations in heterozygous EMS-induced mutants (*cn bw*). All single nucleotide variants (SNVs) identified (ALL) were first filtered against variants present in the isogenized second chromosome (ΔIso). Next, only SNVs affecting coding regions or splice sites were retained (functional). Finally, recurrent background variants detected in multiple sequenced genomes were excluded (ΔEndoA Screen). (**B**) Overview of the number of SNVs on the second chromosome (25 Mb interval) at each filtering step described in (**A**).

© The Author(s)

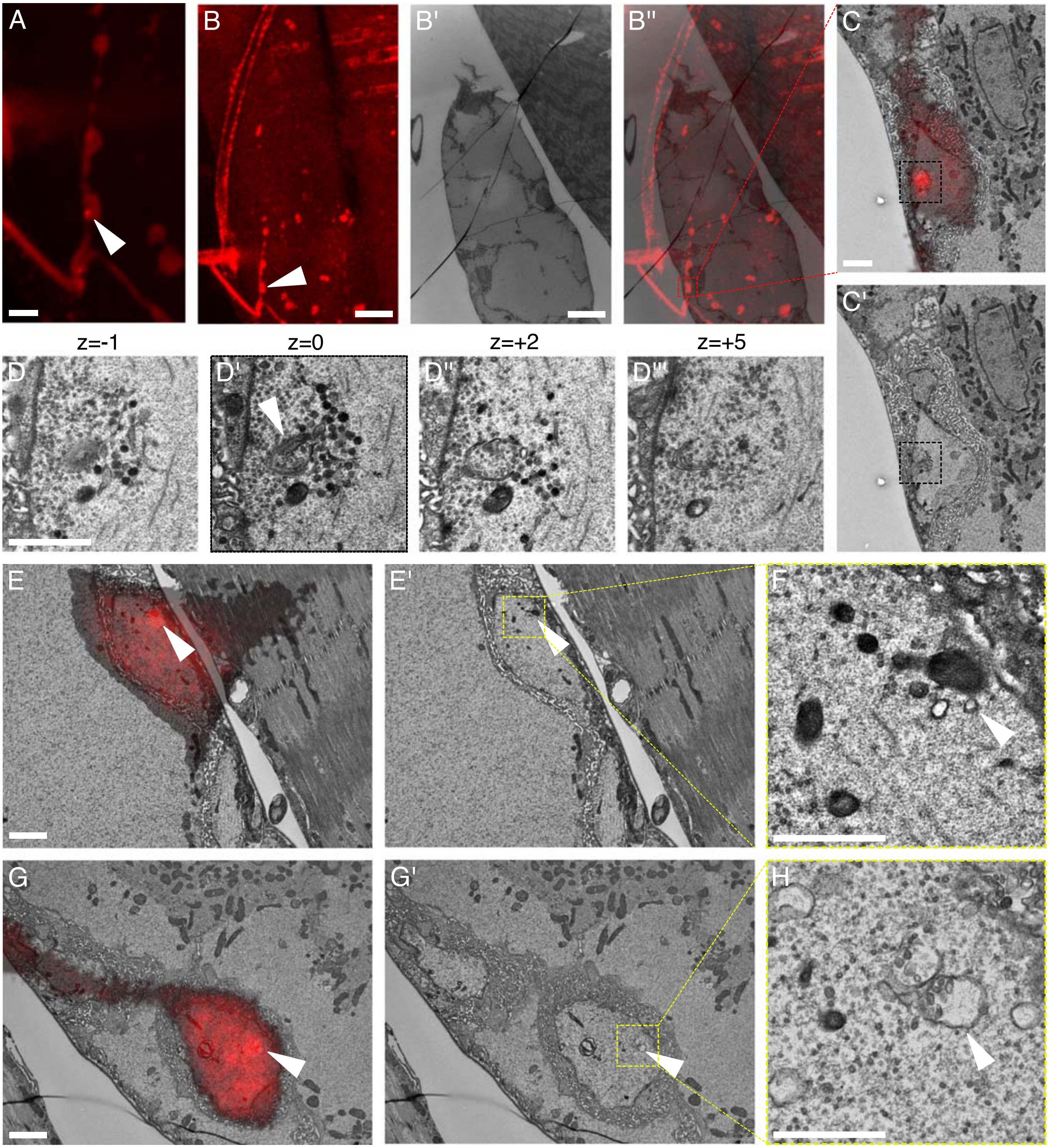

 © The Author(s)

◀ **Figure EV2. CLEM of boutons of *rab39*[KO] animals expressing Atg8-mCherry.**

(**A**) Maximum intensity projection of confocal slices of an example NMJ1 displaying an Atg8-mCherry-positive structure (arrowhead). Scale bar: 5 µm. (**B**) Zoomed-out maximum projection of the same NMJ1 after branding shown in (**A**). Arrowhead indicates the bouton corresponds to the bouton in (**A**). Scale bar: 20 µm. (**B′**) Electron micrograph of the same region as in (**B**). Scale bar: 20 µm. (**B′′**) Overlay of confocal image in (**B**) with the electron micrograph in (**B′**). (**C**, **C′**) Zoomed views of the bouton containing the Atg8-mCherry structure shown as overlay in the red square (**B″**) and EM alone (**C′**). The black square highlights the structure corresponding to the mCherry signal. Scale bar: 2 µm. (**D**–**D′′′**) Single TEM slices showing the putative autophagosomal structure (arrowhead) shown in black square in (**C-C′**), visible in multiple consecutive sections. Scale bar: 1 µm. (**E**, **E′**) Overlay of a fluorescence image section from NMJ2 with the corresponding EM image. Arrowheads indicate the structure corresponding to the mCherry signal. Scale bar: 2 µm. (**F**) Zoom of the structures highlighted in yellow square in (**E′**). Scale bar: 1 µm. Arrowheads indicate the putative autophagosomal structure. (**G**, **G′**) Overlay of a fluorescence image section from another NMJ3 with its corresponding EM image. Arrowheads indicate the structure corresponding to the mCherry signal. Scale bar: 2 µm. (**H**) Zoom of the structures highlighted in yellow square in (**G′**). Scale bar: 1 µm. Arrowheads indicate the putative autophagosomal structure.

© The Author(s)

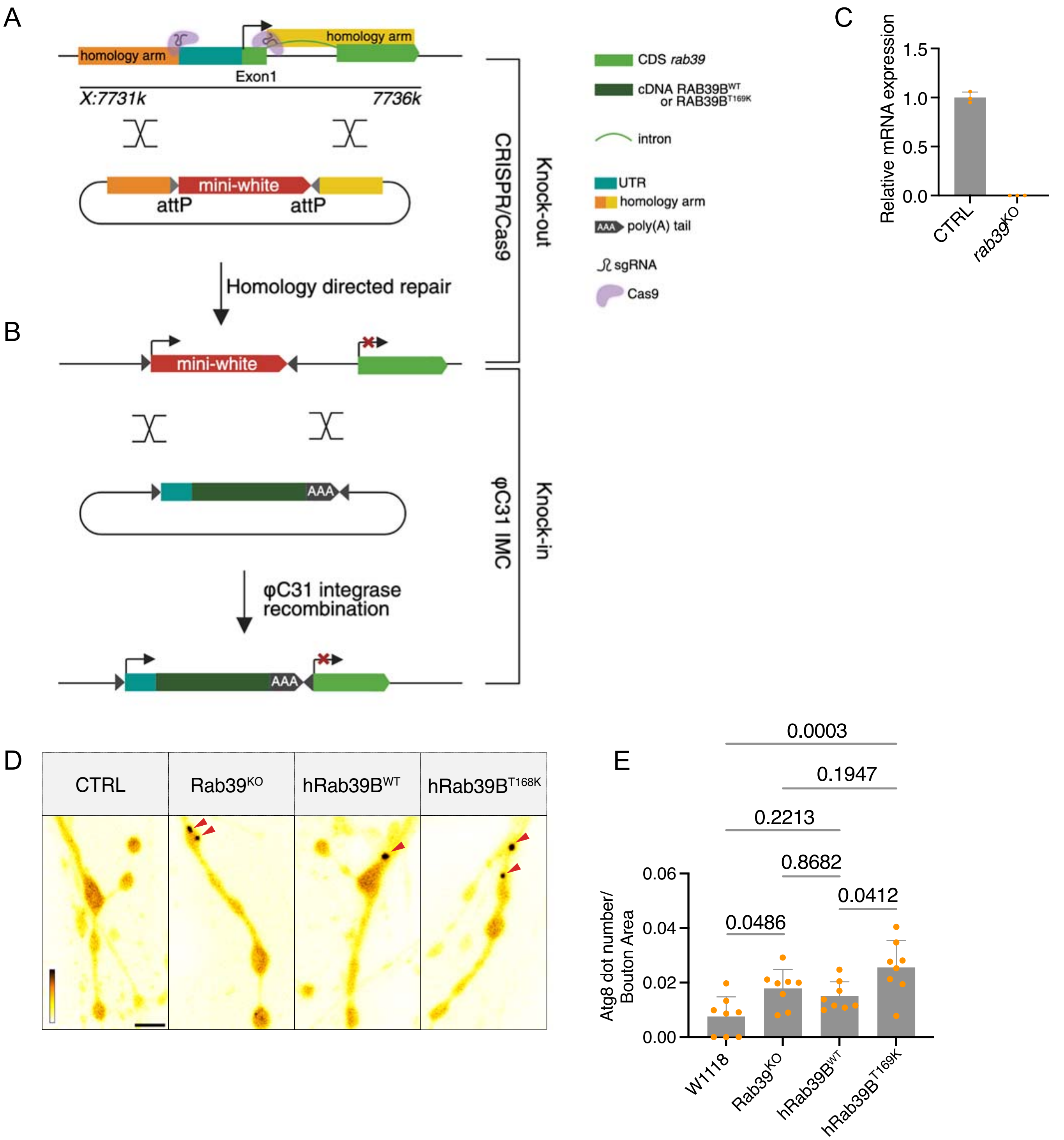

Figure EV3. Pathogenic *RAB39B*^{T168K} mutant mimics *rab39*^{KO} and increases synaptic autophagy.

(**A**, **B**) Schematic of the *rab39* knockout (**A**) and knock-in (**B**) strategy. The first exon of *Drosophila rab39* was replaced with an attP-flanked mini-white cassette via CRISPR/Cas9-mediated homologous recombination, generating a null allele (*rab39*^{KO}). The chromosomal insertion site is indicated. This mini-white cassette was then replaced by the human wild-type or pathogenic mutant cDNA (including a stop codon) to create "human knock in alleles". (**C**) *rab39* mRNA expression levels measured by RT-qPCR. Expression is shown relative to endogenous *Drosophila rab39* transcript levels ($w^{1118}w^{+}$, CTRL). (**D**) Live imaging of genomically expressed Atg8-mCherry in NMJ boutons of control ($w^{1118}w^{+}$, CTRL), *rab39*^{KO}, *RAB39B*^{WT}, and *RAB39B*^{T168K} animals. Fluorescence intensities are shown using the gray value range indicated in ((**D**), CTRL) (323–3045). Red arrowheads mark Atg8-mCherry-positive puncta. Scale bar: 5 µm. (**F**) Quantification of Atg8-mCherry puncta per bouton area from the experiment shown in (**E**). Statistical test: two-way ANOVA with Tukey's multiple comparison test; $n = 8$; error bars: mean ± SD.

 © The Author(s)

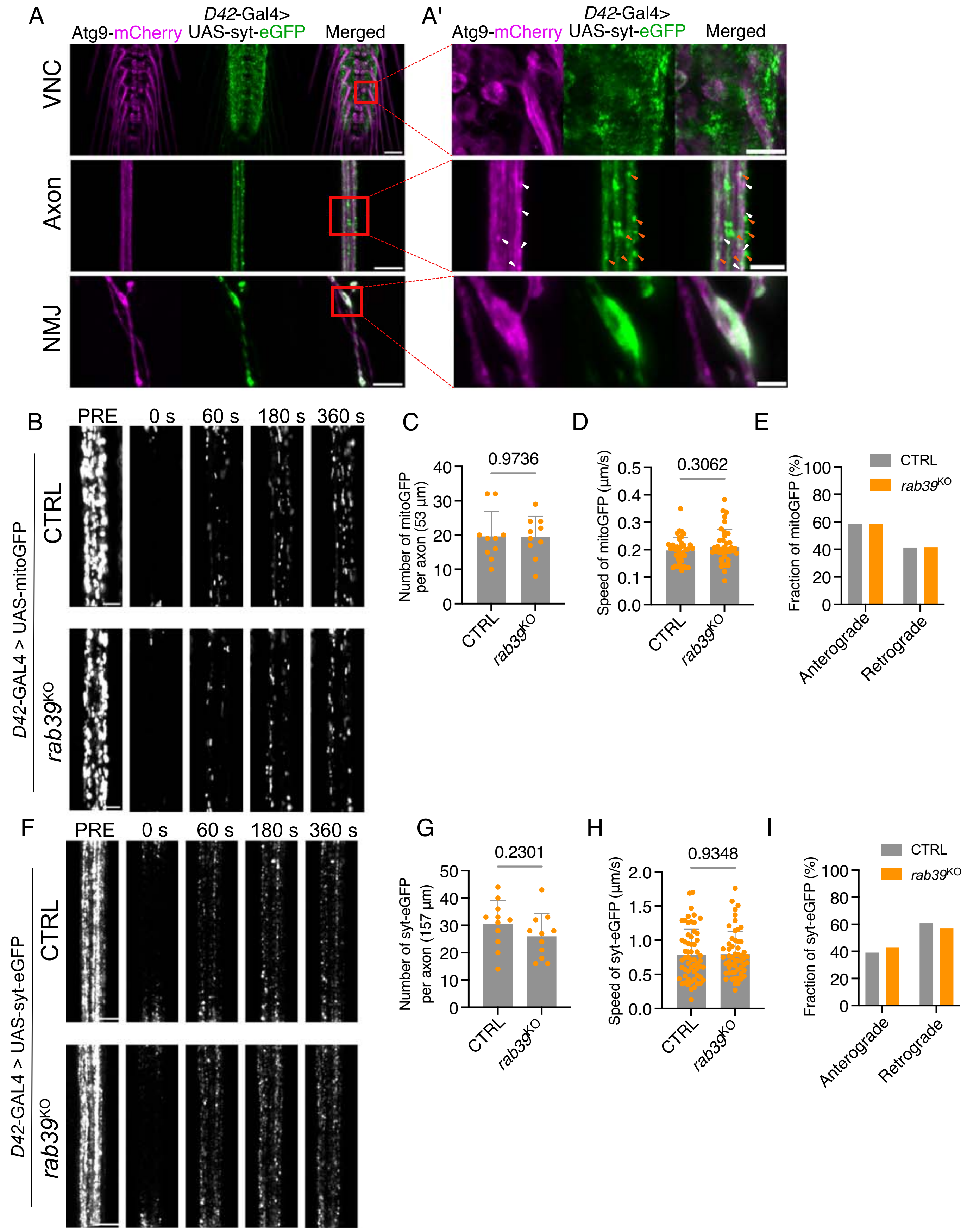
A
A'
Atg9-mCherry
D42-Gal4>
UAS-syt-eGFP
Merged
VNC
Axon
NMJ
B
PRE
0 s
60 s
180 s
360 s
CTRL
rab39KO
D42-GAL4 > UAS-mitoGFP
C
0.9736
Number of mitoGFP per axon (53 µm)
D
0.3062
Speed of mitoGFP (µm/s)
E
Fraction of mitoGFP (%)
Anterograde
Retrograde
F
D42-GAL4 > UAS-syt-eGFP
G
0.2301
Number of syt-eGFP per axon (157 µm)
H
0.9348
Speed of syt-eGFP (µm/s)
I
Fraction of syt-eGFP (%)

Figure EV4. Rab39 does not affect synaptic vesicle or mitochondrial transport.

(**A–A′**) Representative live confocal images showing Atg9-mCherry and Syt-eGFP in the ventral nerve cord (VNC), axons, and neuromuscular junctions (NMJs) of *Drosophila* third-instar w^{1118} larvae. Red squares in (**A**) indicate regions enlarged in (**A′**). White arrowheads mark Atg9-mCherry vesicles; orange arrowheads indicate Syt-eGFP vesicles. Scale bars: 100 µm (**A**) 40 µm (**A′**) (VNC); 40 µm (**A**) 20 µm (**A′**) (axon); 40 µm (**A**), 10 µm (**A′**) (NMJ). (**B**, **F**) Representative pre- and post-bleach images (t = 0 and t = 360 s) of axons expressing *D42*-Gal4 > UAS-mitoGFP (**B**) or UAS-Syt-eGFP (**F**) in control and $rab39^{KO}$ larvae. Scale bars: 5 µm (**B**), 20 µm (**F**). (**C**, **G**) Quantification of mitoGFP- and Syt-eGFP-positive particles per defined axon length (53 µm and 157 µm, respectively). Statistical test: unpaired *t* test; $n = 10$ (**C**), $n = 11$ (**G**); error bars: mean ± SD. (**D**, **H**) Quantification of vesicle speed for mitoGFP ($n = 39$ traces/group from 10 animals) and Syt-eGFP ($n = 59$ [CTRL] and 55 [$rab39^{KO}$] traces from 11 animals). Statistical test: unpaired *t* test; error bars: mean ± SD. (**E**, **I**) Direction of vesicle movement (anterograde vs. retrograde) for mitoGFP (**E**) and Syt-eGFP (**I**) in control and $rab39^{KO}$ axons. Statistical test: Fisher's Exact Test; (**E**) *P* value = 1; (**I**) *P* value = 0.3273 exact counts in Appendix Table S1.

 © The Author(s)

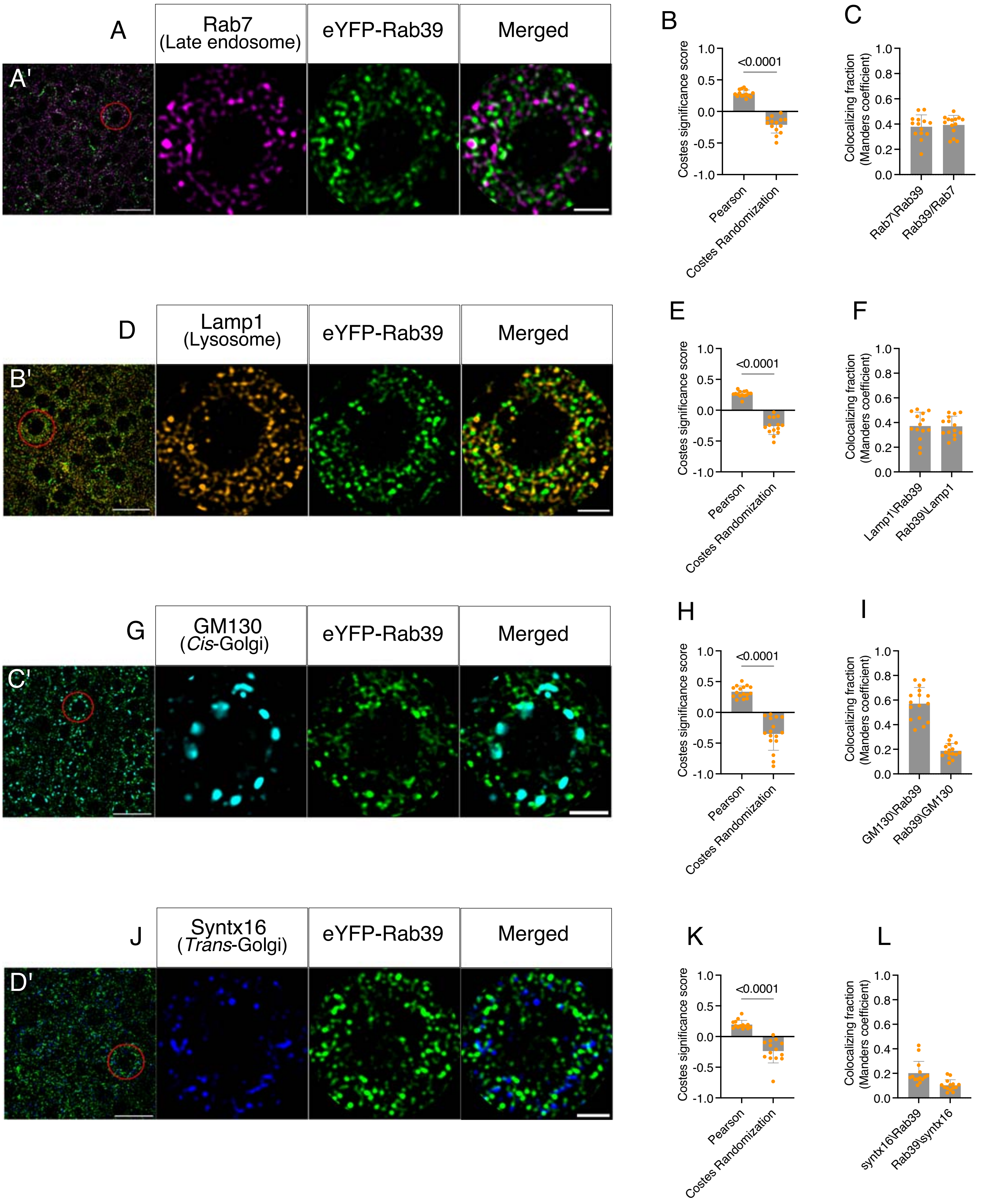
A
Rab7
(Late endosome)
eYFP-Rab39
Merged
A'
B
Costes significance score
<0.0001
Pearson
Costes Randomization
C
Colocalizing fraction
(Manders coefficient)
Rab7\Rab39
Rab39/Rab7
D
Lamp1
(Lysosome)
eYFP-Rab39
Merged
B'
E
Costes significance score
<0.0001
Pearson
Costes Randomization
F
Colocalizing fraction
(Manders coefficient)
Lamp1\Rab39
Rab39\Lamp1
G
GM130
(Cis-Golgi)
eYFP-Rab39
Merged
C'
H
Costes significance score
<0.0001
Pearson
Costes Randomization
I
Colocalizing fraction
(Manders coefficient)
GM130\Rab39
Rab39\GM130
J
Syntx16
(Trans-Golgi)
eYFP-Rab39
Merged
D'
K
Costes significance score
<0.0001
Pearson
Costes Randomization
L
Colocalizing fraction
(Manders coefficient)
syntx16\Rab39
Rab39\syntx16

© The Author(s)

Figure EV5. Rab39 localizes to organelles involved in Golgi–endolysosomal trafficking.

(**A–A′**, **D–D′**, **G–G′**, **J–J′**) Representative Elyra super-resolution images of third-instar larval VNCs showing neuronal cell bodies stained with anti-GFP (eYFP-Rab39, green) and markers for various organelles. Overviews of the VNC are shown in (**A′**, **D′**, **G′**, **J′**). Scale bar: 20 µm; zoomed-in cell bodies in (**A**, **D**, **G**, **J**) are in red circular ROIs in ((**A′**), **D′**, **G′**, **J′**) respectively, Scale bar: 3 µm. Co-stains include anti-Rab7 (late endosome/lysosomes, magenta; (**A**)), anti-Lamp1 (late endosomes/lysosomes, orange; (**D**)), anti-GM130 (cis-Golgi, cyan; (**G**)), and anti-Syntaxin-16 (trans-Golgi, blue; (**J**)). (**B**, **E**, **H**, **K**) Pearson's correlation coefficients for Rab39 and each marker, as well as with Costes' randomization threshold (≥100 iterations). Exact P (**B**) $= 2.913 \times 10^{-8}$; exact P (**E**) $= 3.618 \times 10^{-9}$; exact P (**H**) $= 2.419 \times 10^{-7}$; exact P (**K**) $= 8.892 \times 10^{-6}$. Statistical test: unpaired t test; $n = 14$ (Rab7, Lamp1, Syntaxin-16), $n = 16$ (GM130); error bars: mean ± SD. (**C**, **F**, **I**, **L**) Manders' overlap coefficients indicating the fraction of Rab39 overlapping with each marker and vice versa.

 © The Author(s)
